# Non-Coding RNA-Targeted Therapy: A State-of-the-Art Review

**DOI:** 10.3390/ijms25073630

**Published:** 2024-03-24

**Authors:** Francesco Nappi

**Affiliations:** Department of Cardiac Surgery, Centre Cardiologique du Nord, 93200 Saint-Denis, France; francesconappi2@gmail.com or f.nappi@ccn.fr; Tel.: +33-149334104; Fax: +33-149334119

**Keywords:** microRNAs, long non-coding RNAs, non-coding RNAs, ncRNA therapeutics

## Abstract

The use of non-coding RNAs (ncRNAs) as drug targets is being researched due to their discovery and their role in disease. Targeting ncRNAs, including microRNAs (miRNAs) and long non-coding RNAs (lncRNAs), is an attractive approach for treating various diseases, such as cardiovascular disease and cancer. This seminar discusses the current status of ncRNAs as therapeutic targets in different pathological conditions. Regarding miRNA-based drugs, this approach has made significant progress in preclinical and clinical testing for cardiovascular diseases, where the limitations of conventional pharmacotherapy are evident. The challenges of miRNA-based drugs, including specificity, delivery, and tolerability, will be discussed. New approaches to improve their success will be explored. Furthermore, it extensively discusses the potential development of targeted therapies for cardiovascular disease. Finally, this document reports on the recent advances in identifying and characterizing microRNAs, manipulating them, and translating them into clinical applications. It also addresses the challenges and perspectives towards clinical application.

## 1. Introduction

Non-coding RNAs (ncRNAs) are transcripts that regulate gene expression and protein function. They are generated from the non-protein-coding part of the genome. The two major classes of ncRNA are short microRNAs (miRNAs) and long ncRNAs (lncRNAs). The deregulation of both types of transcript has been linked to every type of cancer that has been studied so far and affects all the major hallmarks of cancer [1,2,3,4,5,6]. Furthermore, they have been associated with intricate biological processes, including the development and function of immune cells, cardiovascular disease, immune disorders, neural development, and neurological diseases [1,2,3,4,5,6,7,8,9,10,11].

Small non-coding RNAs were discovered in the nematode Caenorhabditis elegans 30 years ago [1,3]. These RNAs control gene expression post-transcriptionally. MiRNAs have since been identified in higher eukaryotes and have been found to regulate the majority of mammalian miRNAs [4,5,6]. Nevertheless, the precise number of human microRNAs is still under debate. Out of the 1973 human microRNAs listed in mirBase 22.1 [7], many do not meet the strict curation criteria, such as expression, sequence restrictions, or evidence of productive processing of the progenitor. The number of functional microRNAs in humans ranges from 556 to 758, depending on the source (mir-GeneDB 2.0) [8,9,10]. However, most miRNAs only have effects at high expression levels in tissues, further reducing the proportion of functionally relevant microRNAs. Tentatively, around 150 miRNAs have been implicated in the cardiovascular system.

**Figure 1:**
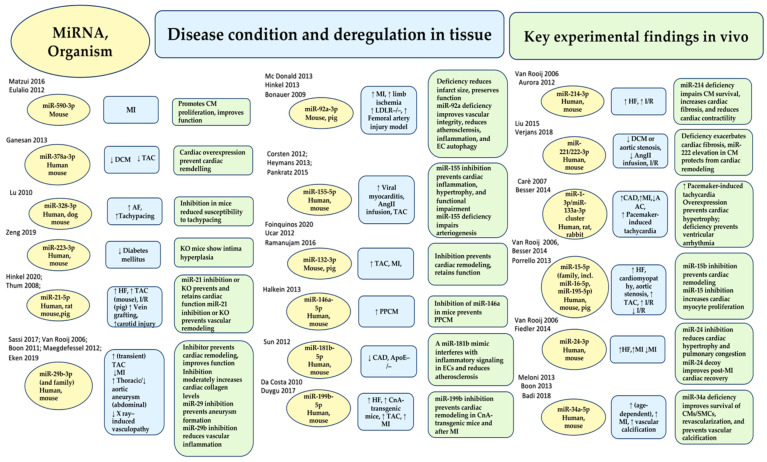
MiRNAs play a crucial role in the cardiovascular system. They are implicated in disease and disease phenotypes, and their effects can be engineered in vivo. The figure shows miRNA species and organisms under investigation (yellow), the disease state and regulation (blue), and key experimental evidence in vivo (green). Abbreviations: AF, atrial fibrillation; AngII, angiotensin II; CAD, coronary artery disease; CnA, human calcineurin subunit A; DCM, dilated cardiomyopathy; HF, heart failure; I/R, cardiac ischemia–reperfusion; CM, cardiac myocyte; EC, endothelial cell; KO, knockout; SMC, smooth muscle cell; MI; myocardial infarction; PPCM, peripartum cardiomyopathy; AAC/TAC, ascending/transverse aortic constriction. From Nappi et al. ref [12]; Refs. [11,12,13,14,15,16,17,18,19,20,21,22,23,24,25,26,27,28,29,30,31,32,33,34,35,36,37,38,39,40,41,42,43,44,45,46,47,48,49] in the figure. Up arrow = increase, Down arrow = decrease.

The development of ncRNA research as a potential therapy for cardiovascular disease has been of particular interest. Among 150 miRNAs, 30 to 35 miRNAs have been extensively studied and confirmed in in vivo experimental models (Figure 1) [11,12,13,14,15,16,17,18,19,20,21,22,23,24,25,26,27,28,29,30,31,32,33,34,35,36,37,38,39,40,41,42,43,44,45,46,47,48,49]. Many of these candidates have already entered clinical development, with several more in the pipeline. Currently, the FDA and/or the European Medicines Agency (EMA) have approved 11 RNA-based therapeutics (Table 1).

## 2. MicroRNA Biogenesis, Stability, and Strand Targeting

**Figure 2:**
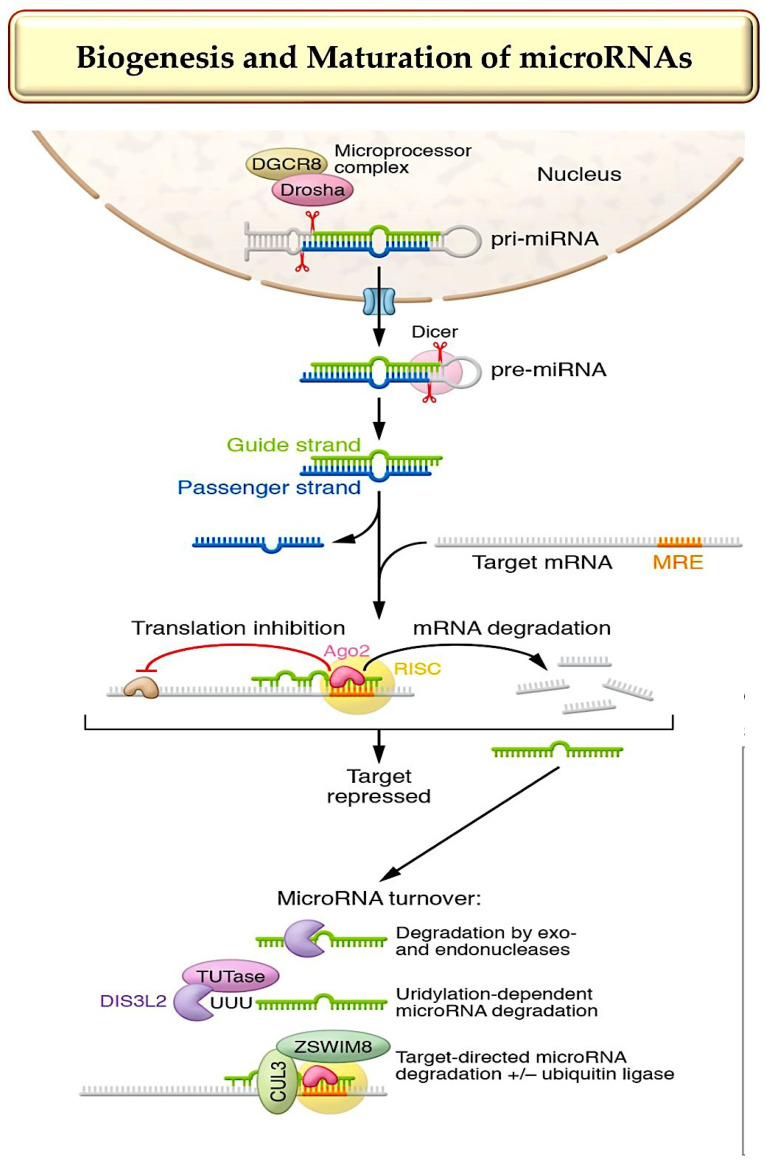
The diagram shows how miRNAs biogenised and function. Three main steps are involved in the synthesis and release of nuclear pre-miRNAs into the cytoplasm, where the final synthesis of activated RNAs is promoted in parallel with the production of miRNA duplexes, RISC complexes, and RNAi: (A) canonical elaboration, functional activation, mechanism of action and degradation pathways of microRNAs are reported. Canonical miRNA biogenesis starts with larger hairpin RNA molecules (pre-miRNAs). These are produced by RNA Pol II transcription of miRNA genes or clusters or occur as part of introns. In the next step, a microprocessor complex, which includes the endonuclease Drosha, the DGCR8 protein, and other factors, cleaves these molecules. Abbreviations: DGCR8, DiGeorge critical region 8 protein; DIS3L2, DIS3-like 3′–5′ exoribonuclease 2; miRNA, microRNA; miRNA duplex, precursor miRNA; RISC complex, RNA-induced silencing complex; RNAi, RNA activation; TDMD, target-directed microRNA degradation; TUTases, terminal uridyltransferases. Refs. [50,51,52].

Transcripts that regulate gene expression and protein function are called non-coding RNAs (ncRNAs). They are generated from the non-protein-coding part of the genome. The two major classes of ncRNA are miRNAs and long ncRNAs (lncRNAs). Excellent reviews have addressed the biogenesis and maturation of microRNAs, as depicted in Figure 2 [50,51,52]. 

The production of long primary miRNA transcripts (pri-miRNA) involves RNA polymerases II and III. The nuclear ribonuclease Drosha and DgCR8 process these transcripts, resulting in the development of a 70 nt long precursor miRNA with a stem-loop structure. The pre-miRNAs are exported from the nucleus by exportin 5 and RangtPase. Finally, the RNase III enzyme Dicer cleaves the pre-miRNAs to yield a mature, double-stranded miRNA. After being processed into 21–22 nucleotide duplexes, one of the strands, called the guide strand, joins the RNA-induced silencing complex (RISC). The other strand, known as the passenger or driver strand, is either incorporated into the RISC or degraded more quickly [53,54] (Figure 2). It is important to note that gene regulation is achieved by degrading the miRNA with RNA helicase and integrating the miRNA guide strand into the RISC. Post-transcriptional gene silencing occurs when miRNAs bind to a nucleotide complementary 3′-UTR, 5′-UTR, or coding region of a target mRNA. miRNAs have a “seed sequence” of two to seven nucleotides at their 5′ end. When the seed sequence binds perfectly with its complement, the mRNA it targets is degraded and deadenylated. However, when the binding is imperfect, which is more common, it results in translational inhibition. RISC facilitates both processes [50,51,52,53,54].

If both strands are preserved, they can have individual functions, as demonstrated for cardiovascular miR-21 and miR-126 [55,56]. There are also microRNA strands that localize to the nucleus and function in unusual ways [56,57]. MiRNA is bound to Argonaute 2 (AGO2) endonuclease and other proteins within RISCs, which are able to regulate both small interfering RNA (siRNA) and microRNA. Mammalian miRNAs require only a seed sequence of seven to eight nucleotides near the 5′ end with full target complementarity, whereas siRNAs require a complete match to their target sequence. Only a few miRNAs rely on these interactions [52,58,59], although additional pairing beyond the seed sequence may help detect targets. MiRNA response elements (MREs), known as microRNA target sites, are primarily located in the 3′-UTR and less commonly in the 5′-UTR or coding regions of mRNAs [6,52]. MicroRNAs have two clearly defined activities: inducing degradation (the predominant activity) or translational silencing of target mRNAs [52].

Non-genetic variants, known as isomiRs, resulting from alternative processing, nucleotide addition, or editing of microRNAs [60,61,62], further enrich the microRNA repertoire. Numerous cardiovascular isomiRs have been identified [63,64]. Their levels vary in different diseases [63]. Different variant and template targetomes were identified for miR-487b-3p and miR-411-5p isomiRs [65,66]. When a microRNA ceases to function, it is enzymatically degraded. MicroRNAs have longer half-lives than mRNAs, but their enzymatic degradation varies depending on factors such as the microRNA strand and sequence, cell type, and trans-acting factors [67,68] (Figure 1). MiRNA targets are also among these factors. Although the mechanistic details of target-directed microRNA degradation (TDMD) have been resolved [52,69,70] and its significance has been demonstrated in vivo [71], identifying mRNAs that engage in TDMD is still challenging.

Long non-coding RNAs (lncRNAs) are transcripts of greater size (>200 nt) that are transcribed in a manner similar to mRNAs but are not processed into protein [72]. lncRNAs include two main classes of functional components: interactor components, which are engaged in direct physical interactions with other nucleic acids, proteins, or lipids, and structural components, which give rise to secondary and/or tertiary 3D RNA structures that control their specific functional relationships [73]. IncRNAs can interact with DNA, RNA, and proteins through base pairing in linear form or chemical interactions in secondary structures. This allows them to function in more variable ways than miRNAs. Many lncRNAs have been identified to have gene-regulatory roles, such as influencing transcription factor binding or epigenetic marks. Additionally, interactions with mRNAs may affect their stability or rate of translation. Likewise, interactions between lncRNA and proteins can influence the stability, activity, or localization of the protein [74,75]. Additionally, circular RNAs, which are similar in length to lncRNAs, are known for their powerful roles as miRNA sponges [76,77].

## 3. ncRNA-Targeting Therapy Is Advancing towards Clinical Use in the Cardiovascular System

**Figure 3:**
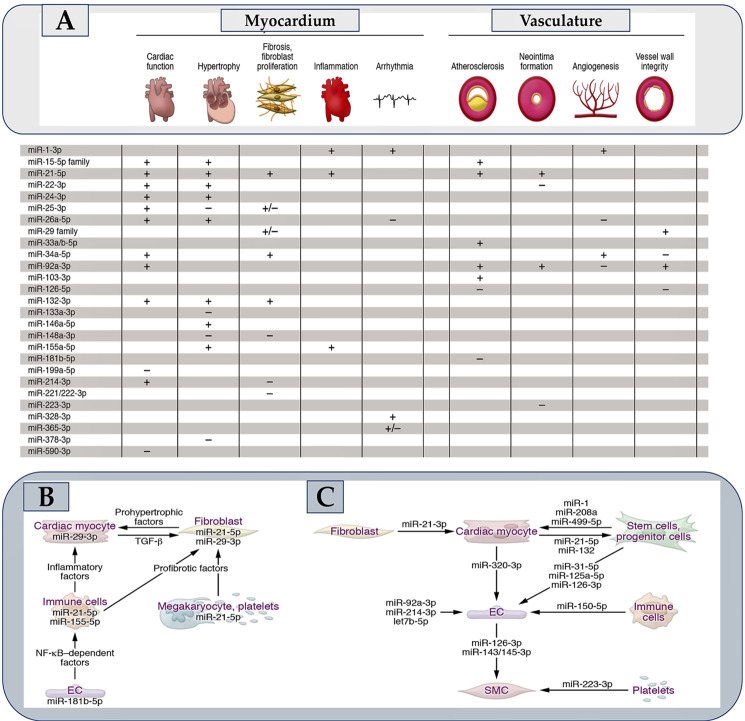
(**A**) summarizes the role of miRNAs in heart muscle and blood vessels. The miRNA that promotes a process is marked with a + sign, and the − sign denotes the miRNA that prevents a pathophysiological process. The microRNAs that either promote or inhibit cardiac function when their levels are elevated or inhibited are described in the respective sections. (**B**) describes microRNAs that regulate the targets responsible for intercellular communication in the cardiovascular system. (**C**) explains the paracrine roles of specific miRNAs secreted within the cardiovascular system. In contrast, the miR-21 core fragment released by endometrial mesenchymal stem cells has cardioprotective effects by promoting cell survival and angiogenesis. Similarly, miRNAs from the myocardium promote the mobilization of progenitor cells in the bone marrow. Platelets carry miR-223-3p, which regulates the differentiation and proliferation of vascular SMCs. Refer to Ref. [50] for a survey of different cardiovascular microRNAs with suggested paracrine activity. Abbreviations: EC, endothelial cell; miRNA, microRNA; SMC, smooth muscle cell. From Laggerbauer B et al. [12,13,14,15,16,17,18,19,20,21,22,23,24,25,26,27,28,29,30,31,32,33,34,35,36,37,38,39,40,41,42,43,44,45,46,47,48,49,50,55,78,79,80,81,82,83,84].

Figure 1 shows 30 to 35 microRNAs that have strong evidence of playing critical roles in cardiovascular health. When manipulated, these miRNAs cause distinct pathophysiological effects in the myocardium or vasculature, as shown in Figure 3A. Some of these activate signaling pathways that cause the secretion of protein factors (see Figure 3B), while others are components of extracellular vesicles, specifically exosomes (see Figure 3C). Alongside the expansion of knowledge, the use of microRNAs for therapeutic purposes in the myocardium and vasculature has significantly increased [78] (Figure 1 and Table 1).

### Investigating Methods for Selecting Therapeutic microRNAs

**Figure 4:**
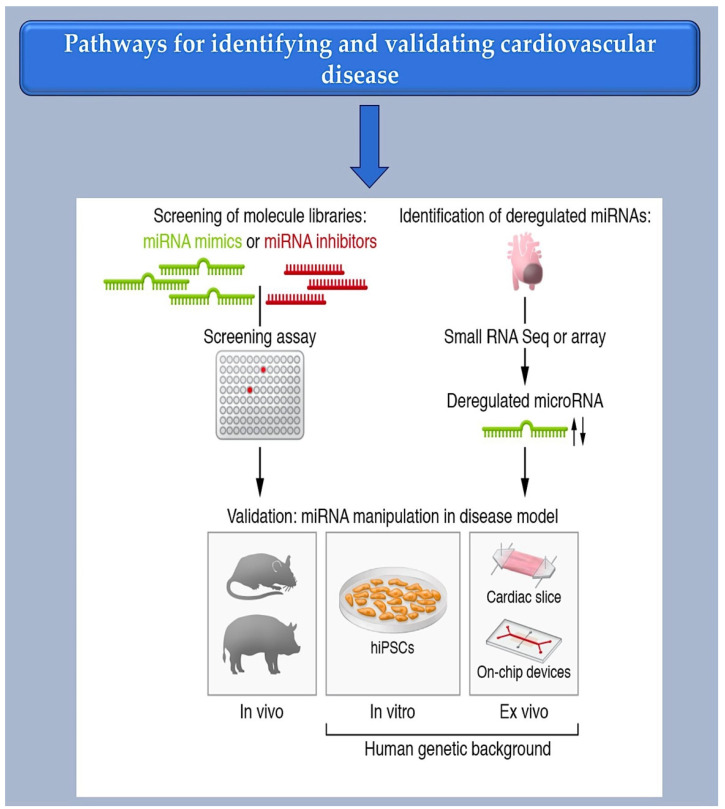
Bioinformatic method for MiRNA characterization through identification of mRNA targets.

As a first step towards the selection of miRNAs by cell culture-based screening, synthetic libraries of miRNA mimics or inhibitors can be generated (see Figure 4). Basically, these approaches either assess the phenotypic effects or show which miRNAs can modulate a desired target. Several studies have assessed functional screening and phenotypic assays in this direction. Functional screening allows miRNAs to be identified in their disease-relevant cellular environment, which is a fundamental advantage of functional screening. Phenotypic assays, such as those for cell survival or morphology changes, are relatively straightforward, quick, and adaptable to high throughput. This has also been demonstrated with cardiovascular cells [14,42,85]. Aside from validating MREs, reporter assays can be used to identify microRNAs that regulate specific miRNAs. This is done by fusing a cDNA for luciferase or a fluorescent protein to the natural 3′-UTR of the miRNA in question and then suppressing or enhancing the expression of the miRNA by introducing an exogenous miRNA mimic or inhibitor. It is important to note that reporter assays typically do not consider miRNAs with MREs located outside of the 3′-UTR. Additionally, these assays do not accurately reflect the miRNA-to-MRE stoichiometries found in physiological conditions, which can lead to errors. MiRNAs with potential therapeutic relevance can be identified by studying miRNA deregulation in disease. Valuable sources for these studies (see Figure 4) are tissue samples from patients or animal disease models.

To unbiasedly detect miRNA candidates, microarrays or small RNA sequencing (small RNA-Seq) are most commonly used. Both methods allow the almost complete discovery of all annotated miRNAs. This means the entire set of miRNAs in a cell type or tissue. Undoubtedly, the cardiovascular system provides a favorable environment for studying miRNA. It offers specific opportunities for the therapeutic development of miRNAs, such as the applicability of non-invasive methods like Doppler sonography or electrocardiography and experimental models that faithfully recapitulate human cardiovascular disease.

The initial stage for therapeutic application involves reprogramming cells for human use. Reprogrammed cells derived from human-induced pluripotent cells (hiPSCs) contain the donor’s genome, making them useful for modeling hereditary cardiovascular disorders. These cells can also form contractile-competent tissues that can be used to test the effects of pharmaceuticals [86]. A human genetic backbone system is provided by a tissue explant culture. Human heart sections or aortic tissue retain their primary organotypic characteristics in culture [87,88] and are amenable to manipulation through virus transduction [89,90], transfection [91], or co-culture experiments [17,92].

By providing an agnostic, unbiased view of the effects of a microRNA across the entire gene expression profile and helping to identify miRNA targets, omics-based methods have become essential for miRNA characterization. One technology that provides relatively easy access to biospecimens is bulk RNA sequencing (RNA-Seq) from dissolved tissue. However, one limitation of tissue use is that aberrant miRNAs may be masked if the cell type in question is overwhelmed by others in which the miRNA is intact. Magnetic cell separation (MACS), fluorescence-activated cell sorting (FACS), or a combination of both can be used prior to RNA-Seq to determine cell-specific expression profiles and reveal low-abundance miRNAs.

Building on this idea, microfluidic separation and genetic barcoding-based single-cell RNA-Seq (scRNA-Seq) provides the chance to determine transcriptomes of individual cells, such as those from healthy [93] and failing human hearts. [94] Various workflows have been developed and validated in a comparative study [95] for the scRNA-Seq of miRnomes, and we can anticipate that one or a few of them will become a widely accepted technical consensus.

Proteome analysis has great potential for diagnosing and analyzing cardiovascular disease alongside RNA-Seq. Patient proteome datasets have been generated from both plasma and tissue [96,97,98]. It is now possible to evaluate changes in the proteome from single cells [99], providing a detailed view of the pathophysiological changes. MiRNAs may have translational repression of their targets, which can be identified by proteomics. Correlating proteomic and miRnomic data can help enrich and clarify RNA-Seq data, leading to a better understanding of miRNA-regulated networks (Figure 4).

**Figure 5:**
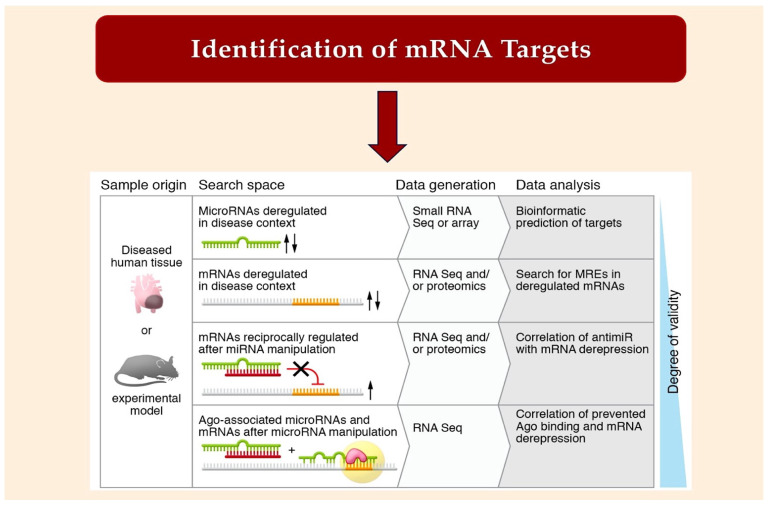
The diagram shows the various methods used to identify microRNA targets. The arrows indicate whether miRNA expression is increased or decreased in the animal model and diseased human tissue. From Laggerbauer B et al. Refs. [23,50,100,101,102,103,104].

Predicting which mRNAs will match a seed sequence has long been the first step in miRNA targeting (see Figure 5), but there are also non-canonical targets [100]. TargetScan predicts multiple mRNA targets for most canonical miRNAs. This is due to the short seed region. Instead of using the miRnome as a search space, one can identify deregulated mRNAs in disease or upon miRNA manipulation (see Figure 5) and analyze the MREs within them. The co-immunoprecipitation of miRNAs with AGO-associated mRNAs and the subsequent sequence analysis can be used to establish a high degree of target accuracy. By comparison of RNA-Seq datasets generated with or without an antimiR, mRNAs that have been derepressed by a miRNA can be delineated as targets ([101] (Figure 5)). Silencing or genetic inactivation of the targets, particularly through mutation of their MREs, are important approaches for validation. It remains to be seen whether there are other parameters, apart from the mere presence of an MRE, that determine targeting [67,102,103]. Most miRNAs are present in a sub-stoichiometric ratio to potential target sites in the transcriptome [104,105], and only those with sufficiently high levels are expected to have a measurable effect on the targetome [103,105]. Evaluating the assumption that certain mRNAs may function as competitive endogenous RNAs (ceRNAs) has provided new insights. Two different hypotheses have been proposed as to how a ceRNA could function as such. One postulates that the RNA must be present in abundance or must incorporate cooperating MREs in close proximity [103,105]. The second theory suggests that a high binding affinity is created by a sequence context beyond the seed match, even when stoichiometry is unfavorable [104]. Evidence supporting the existence of high-affinity sites was obtained by analyzing the ability of anti-miRs to derepress targets [101] (Figure 5). It has been found that dinucleotide motifs in the vicinity of MREs play a role in the affinity of miRNAs [100]. The models agree that usually only widely expressed miRNAs have broad effects on targetomes. An individual target mRNA is usually unable to influence the expression of others. Additional MREs and/or a sequence context contribute to target recognition.

## 4. Translation of Non-Coding RNA-Targeted Therapies through Trials and Approval for Clinical Use

### 4.1. Challenges and Potential Solutions for ncRNA Therapeutics

All the major cancer hallmarks investigated to date have been associated with deregulated levels of both types of transcripts, miRNAs and lncRNAs [106,107,108,109,110]. They have also been implicated in a wide variety of biological pathways, such as the formation and maintenance of immune cells, immune dysfunction [111], neural growth and development, and neurological disorders [112,113,114]. Therefore, therapeutic targeting of naturally occurring ncRNAs presents a promising therapeutic option for a wide range of diseases.

Several RNA-based therapies have been developed. These include antisense oligonucleotides (ASOs), ASO therapeutic circular RNAs (circRNAs), anti-microRNAs (anti-miRs), small interfering RNAs (siRNAs), miRNA sponges, miRNA mimics, short hairpin RNAs (shRNAs) and CRISPR-Cas9-based gene editing. Reviews of these agents can be found in [115,116,117].

### 4.2. Types of RNA-Targeting Therapeutics

RNA-targeting therapies are designed to induce miRNA-like functions, reverse or reduce the levels of a miRNA, or prevent the binding of a miRNA to its target. Chemical modulation is used to enhance the pharmacokinetics and pharmacodynamics of RNA therapeutics, as they are inherently unstable and unable to cross cell membranes due to their negative charge [118,119]. To increase stability, first-generation modifications replace the phosphodiester backbone linkages with phosphothioate (Pt) linkages. Fomivirsen, a first-generation antisense oligonucleotide (ASO) targeting cytomegalovirus (CMV) IE-2 mRNA for the treatment of CMV retinitis, was the first RNA-based therapeutic licensed for clinical application in 1998 (Table 1). Second-generation modifications are designed to improve bioavailability, increase efficacy, and decrease toxicity and immunostimulation by replacing the 2′-O-alkyl group of the sugar moieties with, for example, 2′-O-ME, 2′-MOE or 2′-F. Gapmers are chimeric molecules with a central strand of DNA monomers (to facilitate the RNase H cleavage) surrounded by 2′-modified nucleotides, as 2′-sugar changes tend to repress RNase H activity. In third-generation chemistry, the furanose ring is modified to produce, for instance, locked nucleic acids (lNAs), peptide nucleic acids (PNAs), and phosphoramidate morpholino oligomers (Pmos). All of the currently licensed RNA therapeutics involve chemical adaptation of the second or third generation (Table 1).

ASOs are single-stranded DNA molecules that target specific mRNA and inhibit protein translation. They can interfere with mRNA degradation through the RNase H cleavage or alter pre-mRNA splicing by affecting cis-splicing elements, resulting in exon inclusion or exclusion [120,121].

Small interfering RNAs (siRNAs) can be both single-stranded and double-stranded. They utilize the endogenous miRNA pathway to switch the silencing of a fully complementary mRNA by integrating it into the RNA-induced RISC [122].

Short hairpin RNAs (shRNAs) use the miRNA maturation path followed by Dicer cleavage to form a final double-stranded product prior to RISC loading. Traditionally, shRNAs have been engineered into cells with viral transfer technologies such as adenovirus-associated viruses, retroviruses, or lentiviruses. Bifunctional shRNAs are more effective at knock-down, as they simultaneously generate transcripts with both exact and poor complementarity, triggering both degradation and translational silencing [123]. Currently, two liposomally delivered bifunctional shRNA constructs are undergoing phase I clinical trials: pbi-shRNA eWs/FlI1 [124], which targets the mRNA creating the eWs–FlI1 fusion. The clinical trials NCt02736565 and NCt01505153 are investigating the effectiveness of the protein as a treatment in Ewing’s sarcoma and pbi-stmN1, which targets stathmin 1 mRNA in advanced solid tumors, respectively [125].

MiRNA mimics take advantage of the fact that endogenous miRNAs can simultaneously address multiple mRNAs. MiRNA mimics share an identical structure with an endogenous miRNA, while the passenger chain contains a minor number of mismatches to prevent RISC loading and potential action as anti-microRNA (antimiR) [126]. Two miRNA mimics, mRX34 and mesomiR-1, have been clinically investigated for the treatment of cancer. mRX34 mimics miR-34 [127,128], whereas mesomiR-1mimics miR-16 [129] (Table 2). AntimiRs are antisense oligonucleotides (ASOs) specifically engineered to be completely or selectively complementary to an endogenous miRNA to prevent it from interacting with targeted genes. AntimiRs, when conjugated with cholesterol to improve intracellular delivery, can also be referred to as ‘antagomiRs’. Two miR-122 antagomiRs, Rg-101 (N-acetylgalactosamine-conjugated ASO) (see Table 2) and miravirsen (sPC3649; beta-D-oxy-lNA), have entered clinical trials as novel hepatitis C virus (HCV) therapeutics [130,131] (Table 2). Furthermore, mRg-110, an anti-miR-92a, was evaluated for its ability to selectively activate angiogenesis and enhance wound healing (NCT 03603431). Additionally, the effectiveness of anti-miR-21 (Rg-012) in preventing kidney fibrosis in patients with Alport syndrome was investigated (NCT 03373786) (Table 2).

MiRNA sponges are transcripts that contain multiple miRNA binding sites and are specifically adapted to intercept and sequester miRNAs [132,133]. They can target one or multiple miRNAs [134,135], such as mir-21, miR-155, and miR-221/miR-222 in tumor cells [136], or a whole miRNA seed family, such as miR-181a, miR-181b, and miR-181c [137]. While miRNA sponges have proven to be a useful experimental tool, they have not been translated into clinical applications [138]. MiRNA-masking ASOs are a gene-specific and safe therapeutic strategy that involves masking the binding site of a miRNA within the target gene [139]. This approach is particularly useful in cases where seed-family members have dual effects. Additionally, Tiny 8–10 nt lNAs can be used to silence seed sequences specifically. For instance, a 16 nt oligonucleotide was used to mask the miR-16 binding sites in tYRP1 mRNA, which acts as a miRNA sponge through three non-canonical miR-16 binding sites in its 3′ utR. This restored the tumor-suppressive function of miR-16 in melanoma cells [140,141]. However, miRNA-masking ASOs have not yet been used in clinical settings.

In the last ten years, research into lncRNA therapeutics has increased. However, no lncRNA-targeting therapeutics have yet reached clinical translation. Currently, lncRNAs are being investigated as biomarkers due to their association with various diseases, such as preeclampsia (NCT 03903393), lung cancer (NCT 03830619), and acute ischemic stroke (NCT 04175691). It is probable that lncRNAs will increase the number of RNA-interference (RNAi) and CRISPR targets in the future. Certain types of lncRNAs, such as circular RNAs or natural antisense transcripts, offer promising new treatment options. Currently, the FDA and/or the European Medicines Agency (EMA) have approved 11 RNA-based therapeutics (refer to Table 1) that target gene alterations in the liver, muscle, or central nervous system. These drugs are primarily siRNAs or ASOs that downregulate specific genes or interfere with pre-mRNA splicing, inducing exon skipping or inclusion. While several RNA therapeutics, including newer compounds like miRNA mimics and antimiRs, are currently undergoing phase II or III clinical testing, no therapeutics based on lncRNA have yet reached clinical trials (Table 3).

### 4.3. Benefit of MiRNA-Based Therapeutic

MiRNA-based therapeutics offer two distinct benefits [115,142,143]. First, as opposed to man-made chemotherapeutic agents or ASOs, miRNAs are natively expressed substances in human cells. This means that they have all the necessary machinery to produce and target them downstream (Figure 2). Second, miRNAs act by interfering with several genes involved in a single pathway, thereby eliciting a response that is both broad and specific. The miR-15–miR-16 cluster is an outstanding demonstration of a miRNA working at different scales to impact the same cancer feature by downregulating multiple anti-apoptotic drivers, including BCL-2 and MCL1 [144,145]. Naturally arising miRNAs could, therefore be a viable option to current RNA-based therapies and could potentially enhance therapeutic efficacy compared to synthetic siRNAs or ASOs, which only affect a single target gene.

The varied functions of lncRNAs offer numerous options for their therapeutic targeting. The approach to targeting these lncRNAs should be tailored to their specific mode of action. LncRNA targeting can be achieved through various methods, such as transcriptional inhibition, post-transcriptional inhibition, steric hindrance of secondary structure formation or protein interactions, introduction of synthetic lncRNAs (e.g., circular), and modification of lncRNA genomic loci or modes of expression by CRISPR-Cas9 or CRISPR-Cas13 [146]. The study of natural antisense transcripts (NATs) is a fascinating field: lncRNAs that are transcribed in the antisense (opposite) orientation to the genes they encode thus negatively affect them in cis. Antisense oligonucleotides (ASOs) that target NATs have demonstrated very encouraging preclinical results for gene reactivation in the central nervous system. AntagoNATs successfully increased levels of brain-derived neurotrophic factor (BDNF), a protein that plays a critical role in forming memory [147,148,149].

RNA-based therapeutics face challenges with specificity, release, and tolerance, which have impeded their clinical translation. Specificity issues arise from unintended on-target effects in non-target cells or off-target effects from sequence variations or overdosing beyond endogenous levels. Administering RNA constructs poses three main challenges: the instability of unaltered RNA, the need for endosomal escape mechanisms to ensure effective intracellular release, and the lack of a suitable carrier vehicle for the target organ/cell type. In addition to these considerations, clinical trials are often discontinued due to poor results (Table 2). For instance, Genasense (G3139), a nuclease-resistant ASO that targets BCL2 mRNA, was abandoned due to its poor efficacy [150]. This contrasts with the highly promising application of venetoclax, a small molecule-mediated inhibition of the BCL-2 protein [151]. The problem with tolerability is that RNA structures are recognized by pathogen-associated molecular pattern (PAMP) receptors, such as Toll-like receptors (TLRs), resulting in undesired immune responses. For instance, the miR-34 mimic MRX34 has been associated with serious side effects in five patients, such as cytokine release syndrome, during a multicentre phase I clinical trial in individuals with clinically proven progressive malignancies [127,128]. In contrast, miR-16 restoration therapy in mesothelioma patients (MesomiR-1) [129], management of keloid scars by intradermal injection of the miR-29 mimic remlarsen [152], and initial studies using cobomarsen (anti-miR-155) in cutaneous T-cell lymphoma have not shown life-threatening toxicities [115,116]. These findings suggest that with the appropriate assessment of toxicities and improved delivery methods, miRNAs may be suitable for therapeutic development.

Potential approaches to overcome the disadvantages experienced in the clinical application of RNA-based therapeutics may be a necessary way to better understand the true role of non-coding RNA in therapeutics. The hurdles of immune responses, low specificity, and nonspecific delivery, with a particular focus on miRNA- and lncRNA-based therapeutics, represent another point of concern that must be elucidated. RNA-based interventions can be used to treat diseases caused by pathogenic RNAs, including those derived from the human genome and xenogenomes such as RNA viruses (e.g., SARS-CoV-2). This section provides an overview of recent and promising preclinical and clinical advancements in the field [153].

### 4.4. Oligonucleotide-Based Therapy Insight

Antisense oligonucleotides (ASOs) are used as single strands to inhibit microRNA (miRNA) and do not integrate into the RNA-induced silencing complex (RISC). It is important to note that diseases cause significant changes in endogenous microRNA levels, ranging from 3- to 4-fold up to 30-fold deregulation [154]. These changes, either alone or in combination with other microRNA regulations, can have a dramatic impact on the targetomes and disease phenotype [155,156].

MiRNAs that are transcribed together in a cluster can work together, as demonstrated by the miR-106b~25 cluster [157]. However, individual microRNAs can also regulate multiple levels of a cellular process. Examples of such regulation include miR-378a-3p, the miR-29 family, and miR-365-3p [16,17,91,158]. In summary, microRNAs combined and multilevel activities enhance their ability to modify diseases. Reverse complementary base pairing is often used to target and inhibit most of the oligonucleotides in therapeutic development. These are ASOs that cause RNase H cleavage, morpholinos that mask translation initiation or splicing regions, siRNAs, and microRNA inhibitors [154]. Currently, there are 10 approved drugs based on siRNAs or other ASOs, with several more in clinical studies. Inclisiran, a siRNA that reduces LDL cholesterol and prevents atherosclerosis, can be considered the first-in-class ASO for treating cardiovascular disease [159].

The most advanced microRNA-based drug candidates for the treatment of hepatitis C are anti-miRs targeting miR-122-5p, known as miravirsen (Anti-miR-122/SPC3649), and anti-miRNA/miRNA-122, known as RG-101 (Table 2). However, due to the exceptional efficacy of other drugs and the gradual development of viral resistance, their medical need has diminished [160]. Nonetheless, these anti-miRs have demonstrated the feasibility of microRNA-based therapy in patients. At the beginning of 2002, 19 clinical trials involving microRNA-based therapeutics were concluded or underway. Two other trials involving miR-103/107-3p (RG-125/AZD4076) and one involving miR-155-5p (cobomarsen/MRG 106) were discontinued or stopped by the sponsor for strategic reasons (Table 4). It is important to underline that the molecules that have been removed are those included in the second-generation chemical modification. These miRNAs have commonly been used in RNA-based therapeutics and were designed to reduce the immunostimulatory potential of synthetic RNA therapeutics. 2′-ribose modifications on siRNAs, such as 2′-F, 2′-O-Me, and 2′-H, can abrogate TLR stimulation, particularly when applied to uridines within GU-rich sequences [161,162,163]. For example, the preliminary results in terms of effectiveness and security have been promising for the 23 nt ds miR-34a mimic (MRX34). Although MRX34 demonstrated potent anti-tumor activity in preclinical studies [164,165], the first-in-human MRX34 clinical trial was discontinued due to immune-related adverse events in five patients. These adverse effects consisted of systemic inflammatory response syndrome, cytokine release syndrome, enterocolitis, hepatic failure, hypoxia, and respiratory failure [128]. The immune effect of miR-34a was unexpectedly strong. Preclinical studies in mice did not show any immunogenicity, as assessed by IL-1β, IL-6, and TNF secretion [166]. A study in which the same administration vehicle was used to deliver ssDNA molecules (PNT2258) also failed to show any evidence of immune stimulation [167]. Notably, this suggests that the delivery vehicle is not the cause of immune stimulation [167]. MRX34 is most abundant in the liver, bone marrow, and spleen in non-human primates [168]. A downregulation of miR-34a target genes in white blood cells and an increase in miR-34a levels in tumor tissue were observed in the pharmacodynamic analysis of patients in the phase I study. However, whether gene silencing or immune-mediated anti-tumor activity was responsible for the three patients who responded to MRX34 therapy (4% response rate) is uncertain [128]. It is worth noting that miR-34a targets the well-known immunotherapy target programmed cell death 1 ligand 1 (PDL1) [169], which has been implicated in the observed responses.

Apart from ASOs, there are currently no microRNA mimics or over-expressions being applied for cardiovascular indications that are close to clinical trials.

## 5. Development of microRNA-Based Cardiovascular Therapeutic Approaches in Clinical Trials

Table 4 shows the clinical trials of the miRNAs with therapeutic potential in cardiovascular disease.

The impact on the cardiovascular field appears to be less than expected, although several miRNA-targeted therapeutic developments in other indications have been discontinued (e.g., miravirsen, RG-101, cobomarsen, and AZD4076). Preclinical and clinical evidence provides valuable information for the design and performance of miRNA-targeted cardiovascular therapies, even for those oligonucleotides that have been discontinued. These miRNAs have been extensively studied in both laboratory and clinical settings (see Table 2). Of particular interest is the miR-132-3p inhibitor (CDR132L) being developed for the treatment of heart failure. CDR132L could become the first miRNA-targeted drug for cardiovascular therapy and is currently scheduled for phase II testing (see Table 2).

Apart from the ASOs, there are currently no microRNA mimics or over-expressions being applied for cardiovascular indications that are close to clinical application. It is important to note that the timing and dosage of microRNA-boosting therapy is critical, as evidenced by the complications observed in the MRX-34 anti-tumor trial [128] and the deleterious impact of both also prolonged miR-199a and miR-92a expressions in the mouse models [183].

### 5.1. Evaluating the Tropism of Oligonucleotides: Open Questions and Major Challenges

**Figure 6:**
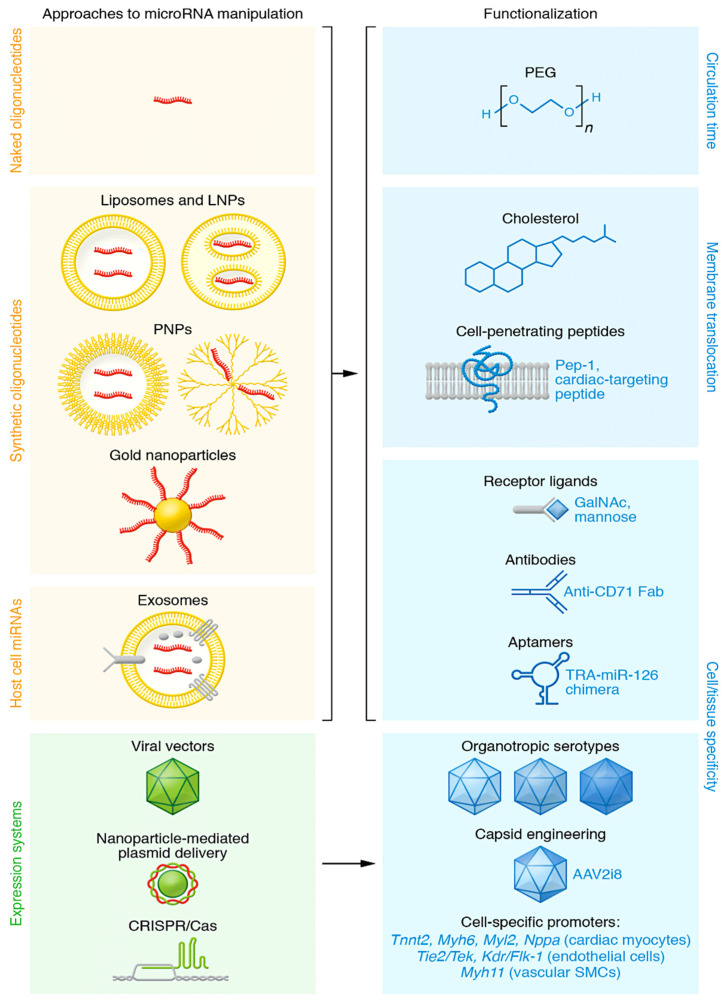
The diagram shows molecular vehicles for microRNA modulators and how they can be functionalized. The use of modified nucleotides in synthetic oligonucleotides improves nuclease resistance, allowing for their use as “naked” molecules. Cell entry via endocytosis can be improved by embedding them in liposomes, lipid nanoparticles (LNPs), or polymer-based nanoparticles (PNPs). Metallic particles, including gold, have also been used to carry oligonucleotides and plasmids. Exosomes carrying microRNA can be obtained from natural sources or designed for better microRNA loading or cell targeting [104,105]. Oligonucleotides or their delivery vehicles can be further function-modified by coupling to improve their circulation time (e.g., by PEGylation), membrane penetrance (e.g., cholesterol, cell-penetrating peptides), or cell- or tissue-specific delivery (e.g., by coupling to receptor ligands, antibody fragments, or aptamers). The transferrin receptor aptamer (TRA) is an example. The expression or genetic inactivation of microRNAs or their targets can be achieved using viral vectors, in particular adeno-associated virus (AAV). AAV engineering [184,185] can improve transduction and/or tropism, and the use of cell type-specific promoters can further enhance the process. Gene expression in cardiac myocytes is denoted by exemplary promoters such as Tnnt2 (cardiac troponin T2), Myh6 (myosin heavy chain 6), Myl2 (myosin light chain 2), and Nppa (natriuretic peptide A). Similarly, in endothelial cells, protein tyrosine kinase Tie2/Tek and Kdr/Flk-1 (kinase insert domain receptor/fetal liver kinase 1) are used as promoters. In vascular smooth muscle cells, Myh11 (myosin heavy chain 11) is used as a promoter. From Laggerbauer B et al. Refs. [50,187,194,195,196,197,198,199,200].

The challenge of achieving efficient oligonucleotide concentrations in target tissues or cells has led to a number of strategies that are summarized in Figure 6. Many of these hold considerable hope for cardiovascular applications. Oligonucleotides, because of their hydrophilic properties, do not readily cross membranes. Additionally, their distribution into cardiovascular tissue may be overtaken by renal filtration [60]. In addition, the endothelium fenestration in the liver and the presence of high levels of monocytes in the spleen and bone marrow decrease the cardiovascular accessibility of oligonucleotides [184]. In the myocardium, this results in relatively low cellular incorporation [185], although this process seems to be enhanced in pathological conditions [186]. Oligonucleotides face the problem of being sequestered in endosomes after endocytosis, from which they must emerge in order to deliver to their targets [184]. LNA antimiRs can partially overcome these hurdles by penetrating membranes as “naked” molecules [187]. Indeed, many cardiovascular studies are conducted without the formulation of antimir, as shown in Table 5.

**Table 5:** Composition, mode of delivery, and dosage schedules of selected synthetic inhibitors or mimics of microRNAs.

Synthetic Molecule ϕ Ref.	Organism	Composition	MoD	Dosage Schedules
**AntimiRS**				
LNA-antimiR-29 [17]	Mouse	Saline	I.V	20 mg/kg, 1 daily dose for 3 days, starting d1 after surgery.
LNA-antimiR-15b [188]	Mouse	Saline	i.v. via catheter	Up to 33 mg/kg, 1 dose 3 days after AngII infusion.
LNA-antimiR-26a or miR-26a mimic [189]	Mouse	Matrigel	s.c	1 × 10^6^ cells/mL Matrigel transfection: 30–100 nM oligonucleotide/5 × 10^4^ cells
LNA-antimiR-15 [190]	Mouse	Saline	s.c	2 doses with 5 mg/kg each (2–3 days before TAC, 3–4 days after)
LNA-antimiR-26a [190]	Mouse	Not candidate	i.v	24 mg/kg, 1 dose 24 h before MI
LNA-antimiR-15b [188]	Pig	Saline	i.v	Up to 3.3 mg/kg
LNA-antimiR-22 [191]	Mouse	Hydrogel	Perivascular	2.5 nmol Injection concomitant with surgery
LNA-antimiR-21 [18]	Pig	Saline	i.v	10 mg each on d5 and d19 after MI
**Antagomirs**				
Antagomir-199b [38]	Mouse	Saline	i.p	0.05–80 mg/kg
Antagomir-25 [192]	Mouse	Saline	i.p	80 mg/kg, 1 daily dose for 3 days, starting day 1 after surgery
Antagomir-21 [18]	Mouse	Saline	i.v. via catheter	80 mg/kg, 1 daily dose for 2 days, starting d1 or d21 after surgery.
Antagomir-29b [117]	Mouse	Saline	i.p	80 mg/kg, 1 daily dose for 2 days, starting d1 or d21 after surgery
Antagomir-146a [193]	Mouse	Saline	Not indicated	8 mg/kg d2 before delivery and d1, d3, and d7 after surgery

Table 5 displays the therapeutic dosage and composition administered in the animal model. Abbreviations used in this text include the following: MoD, mode of delivery; AngII, angiotensin II; i.v., intravenous; s.c., subcutaneous; i.p., intraperitoneal.

However, numerous strategies have been devised to enhance the circulation duration, membrane transport, intracellular delivery, or tissue tropism of oligonucleotides [154]. Lipid-based, polymer-based, hybrid, or metal-based nanoparticles are utilized as vehicles for oligonucleotides [194]. Additionally, oligonucleotides are conjugated with polyethylene glycol (PEG) to delay the clearance of agents, which is a common approach (Figure 6). Cholesterol can be attached not just to oligonucleotides to help them cross the membrane but also conjugated to nanoparticles. Cell-penetrating peptides (CPPs), one of which is a cardiac-targeting peptide, have demonstrated efficacy in experimental models of cardiovascular disease in vivo [201,202]. A CPP conjugate of eteplirsen is currently being investigated in a phase II clinical trial for the treatment of Duchenne muscular dystrophy (ClinicalTrials.gov NCT04004065).

Coupling oligonucleotides or microRNA vehicles to receptor ligands or other cell-targeting molecules is expected to result in the most effective cellular tropism [203] (Figure 6). To provide coupling partners for oligonucleotides, molecules must bind to cell-surface proteins, and they must not interfere with the translocation or activity of the drug or cause adverse reactions. As part of this strategy, a therapeutic approach involved using a siRNA coupled with a CD71 Fab′ fragment to target the heart and skeletal muscles in mice. This approach was found to be effective in treating muscular dystrophy [204]. Other promising candidates are centyrins. These are fibronectin-3 derivatives that can be designed for specificity and affinity and coupled to oligonucleotides [205]. In mice, a folate-coupled antimiR against miR-34-3p preferentially affects tumors [206]. Oligonucleotides linked to N-acetylgalactosamine (GalNAc), a natural ligand of the asialoglycoprotein receptor 1, which is highly expressed in liver cells, are clinically advanced, making them ideal for liver-targeted therapy (Table 2). Other sugars may also be useful for cell-specific oligonucleotide release, such as mannose, whose receptor is mainly expressed on macrophages. In addition, aptamers have been evaluated in conjunction with using siRNAs [207]. One aptamer was found to enhance miR-126-3p release by binding to the transferrin receptor [208].

Adeno-associated viruses (AAVs) are a type of viral vector used to transport genetic information. They are known for their organotropic serotypes, which can be further optimized through capsid engineering. A specific example is AAV2i8, a chimera of an AAV2 inner loop mutant and AAV8 [195,196], which is particularly effective in transducing myocytes [209]. This construct has been used to drive constitutively active inhibitor-1 expression in a porcine model of cardiac ischemia [210] and is presently being evaluated in a phase I clinical trial. (ClinicalTrials.gov NCT04179643). More specifically, targeted evolution has recently produced AAVs with superior muscle cell specificity and transduction efficiency [211]. The use of specific promoters for gene regulation in different cardiovascular cell lines (Figure 6) broadens the range of possibilities.

In addition to the benefits provided by viral vectors, molecular genetic tools like CRISPR/Cas plasmids can also be introduced non-virally (see Figure 6), e.g., by transfection. It is yet to be determined whether the delivery of plasmids for non-coding RNA (as demonstrated for a circRNA construct in Figure 6) will be effective for microRNA expression [197].

### 5.2. Assessing How to Manage

Tissue-specific oligonucleotide targeting has not yet reached late-stage therapeutic trials. Therefore, the route of administration remains important to improve efficacy. In experimental models and in the phase Ib trial of antimiR-132, an intravenous infusion of oligonucleotides is the most commonly used route (Table 5). However, it is important to note that intravenous injection quickly dilutes the drug.

Additionally, the fenestration of certain non-cardiovascular tissues exacerbates this issue. While intraperitoneal injection has been used in cardiovascular preclinical studies [28,212,213], and intracardial injection has been applied in rodents [13,214], the risks associated with either administration method make them unsuitable for use in humans. However, it is important to note that intravenous injection rapidly reduces the concentration of the drug. Additionally, the fenestration of certain non-cardiovascular tissues exacerbates this issue. While intraperitoneal injection has been used in cardiovascular preclinical studies [28,193,212], and intracardial injection has been performed in rodents [13,214], the risks associated with both administrations preclude their use in humans. Oligonucleotides have been successfully applied subcutaneously or intradermally in cardiovascular studies involving mice [186,189,215] and monkeys [215]. Due to their minimally invasive nature and advantageous pharmacokinetics [185], they are favorable for microRNA-based drugs (refer to Table 5). It is important to note that skin reactions at the injection site were frequently observed in clinical studies [216]. This aspect will be further discussed in relation to immunogenicity. To exploit the benefits of local drug delivery with a low risk of tissue damage, several studies have used device-based methods. Coronary catheterization, which is now a clinically routinized procedure, has been used to administer microRNA drugs in both small [18] and large animals [15,27,194].

### 5.3. Assessing Dosing

In the in vivo models of cardiovascular disease, the majority of microRNA mimics or inhibitors are administered in sequential doses within a few hours to a few days after induction of the disorder (Table 5). Therapeutic effects of LNA antimiRNAs were observed within 2 or 3 days in cases where they were tested [189,191,217]. MicroRNA modulators have enhanced nucleus retention and display characteristic half-lives of 3 weeks in cardiac tissue. This suggests that the duration of action is at least 18 to 46 days in mice [13,17,18] or 28 days in pigs [28], allowing for the endpoints of these studies. AntimiR-loaded nanoparticles showed an interesting sustained effect of about 4.5 months. However, it is unclear whether this is due to the preparation method [218]. With only one or two subcutaneous injections per year, the siRNA drug inclisiran provides therapeutic effectiveness. The design and evaluation of miRNA drugs with similar properties and pharmacokinetics should be stimulated by this promising result.

### 5.4. Assessing the Risk of Adverse Effects

- Understanding immune reactions

When considering RNA-based therapies, there are three main sources of potential immunogenicity: (a) the nucleotide portion or its chemical modification, (b) the drug moiety, and (c) the vector used to deliver the overexpression. Unfortunately, a phase I trial of a miR-34 mimic for the treatment of resistant cancers was discontinued due to lethal immune reactions. [128] It is unclear which of the drug compounds triggered the immune reactions. Similarly, immune responses observed with specific ASOs [219] have not been entirely elucidated. Table 4 shows promising safety data from many other clinical studies in contrast to these occurrences. The innate immune response identifies oligonucleotides as pathogen-associated molecular patterns (PAMPs). Toll-like receptors (TLRs) are a class of family of pattern recognition receptors (PRRs) that sense double- and single-stranded oligonucleotides. Replacement of specific nucleotides can decrease the immunogenicity of a siRNA without compromising its potency [220]. Similarly, naturally occurring modifications to nucleosides aid in evading TLR recognition [221], such as 2′O-Me [222] or LNA modifications [223]. The existence of this feature is, therefore, the best-known mechanism to account for immunotolerance to LNA antimiRs.

Nanoparticle packaging can protect oligonucleotides from PRRs, and PEG is utilized in oligonucleotide medications for this reason, in addition to the prolonged circulation benefit. However, it is important to note that PEG can lead to the induction of antibodies, which have been implicated in serious adverse events in one case [224]. Therefore, the potential safety issues related to PEG should be taken seriously despite the long list of well-tolerated and approved PEGylated drugs. Thus, although there is an availability of a long list of well-tolerated and licensed PEGylated agents, the potential for PEG-related adverse events must be taken into consideration. Viral vectors have the potential to cause side effects and to induce neutralizing antibodies (if not present a priori) because of their immunogenicity. In addition, the use of immunosuppressive drugs is a common feature of licensed virus-based gene therapies.

- Understanding toxicity

Chemical modifications to oligonucleotide drugs could potentially cause toxicity through both sequence-dependent and sequence-independent mechanisms. The strong protein binding of certain gapmers has been suggested to be responsible for the latter [225]. In contrast to gapmers, microRNA mimics or antimiRs have been reported to have a more consistent profile of variation at the 2′-ribose position. The reason why most preclinical and clinical studies on microRNA mimics or antimiRs have reported excellent levels of safety and tolerability [160,170,226] may be partly related to this. It has been reported that high doses of antimiRs (>80 mg/kg), regardless of their chemical modification, exhibit sequence-dependent toxicity [212]. It should be noted that antimiRs in clinical translation are used at significantly smaller doses and with a more benign risk profile (see Table 3). This toxicity may be due to antimiRs inhibiting AGO targeting, thereby freeing other microRNAs to gain access to the RISC [227]. Similarly, an overabundance of microRNA mimics can prevent endogenous microRNAs from entering the RISC [228] or binding non-specifically to RNAs. It is unclear whether this is the cause of the unexplained complications observed in the miR-34-mimic study [128].

- Understanding tumorigenesis

Several microRNAs implicated in cardiovascular diseases have also been suggested to play a role in cancer [142]. It is now evident that heart failure and cancer share pathophysiological mechanisms [229], prompting the question of whether targeting specific microRNAs could be advantageous in treating both conditions. Anti-miRs targeting miR-21-5p, miR-146a-5p, or miR-155-5p not only have therapeutic cardiovascular effects but also prevent tumor growth in respective mouse models [180,230,231]. Some evidence supports this hypothesis. While some members of the genetic cluster, such as miR-92a-3p, appear to have less significance in cancer, others have been found to be critical [232]. It is important to note that continuous and uncontrolled cardiac overexpression of miR-199a in pigs resulted in the formation of weakly differentiated myoblasts, leading to fatal arrhythmia [183]. Therefore, this must be taken into consideration during the risk assessment. Based purely on cell culture tests, expression data, or target plots, a number of miRNAs with well-documented cardiovascular function have been ascribed an oncogenic or tumor-suppressive function. Therefore, the risk of tumorigenesis should be evaluated by long-term assessments in animal models and by examination of tissues outside the cardiovascular system.

## 6. Looking Ahead

A clear indication of the advances made in the last decade is the increasing number of clinical trials targeting microRNAs, leading to the first clinical trial of an anti-mRNA in cardiovascular therapeutics. The existence of uncharacterized microRNAs suggests a broader range of potential disease treatments and applications for microRNA therapeutics than is currently apparent. As stated in a critical review of the large body of descriptive literature on microRNAs [233], the field is challenged to rigorously confirm the function of microRNA candidates. The key to improving the therapeutic design and decreasing the risk of attrition will be the combination of miRNA manipulation in disease models, omics technologies, and thorough preclinical evaluation. While the creation of synthetic oligonucleotides has overcome significant challenges, the administration of these molecules remains a major issue. This is particularly relevant for cardiovascular tissue. Cardiovascular tissue does not efficiently incorporate oligonucleotides. It would be desirable, once the pharmacokinetics of oligonucleotides have been further improved, to eliminate the need for specific delivery methods, such as local catheter-based administration. It is also promising to tailor oligonucleotides not only for enhanced cellular absorption but for enhanced cellular targeting. This area is currently underdeveloped and will require a huge investment in ligand screening and chemical binding to oligonucleotides, as well as the development of assays to determine cellular oligonucleotide levels.

The discovery of circular lncRNAs (circRNAs) as miRNA sponges expands the potential for blocking the function of oncogenic RNAs. The ciRS-7 transcript was the first characterized circRNA, highly expressed in the human brain and containing over 70 conserved miR-7 binding sites [234]. CircRNAs are particularly abundant in brain tissue [235], while they are mostly downregulated in cancer tissue [236]. The low transcription but high stability of these transcripts in rapidly proliferating tissues may explain their dilution [237]. CircRNA deregulation has been associated with various diseases, such as cancer, diabetes, and atherosclerosis [236]. Therefore, endogenous or synthetic circRNAs have the potential to be used as effective and stable miRNA sponge therapeutics. The study referenced in [238] demonstrated that foreign circRNAs can trigger an immune response mediated by introns through the PAMP (pathogen-associated molecular pattern) receptor RIG1 (retinoic acid-inducible gene I). Endogenous circRNAs with N6-methyladenosine (m6A) modifications were able to reduce immune stimulation [239]. Synthetic circRNAs have great potential as miRNA sponge therapeutics after further characterization of their immunogenic properties. Synthetic circular mRNAs are also being explored to reconstitute protein expression and were found to be less immunogenic (no RIG1 or TLR stimulation in vitro) and highly translated [240].

## Figures and Tables

**Table 1:** FDA- and/or European Medicines Agency-approved RNA therapies.

Treatment	Type	Amendment & Product Delivery	Mode of Delivery	Destination Site	Disease	Target Gene and Route	FDA and/or EMA Approval Year
Lumasiran (Oxlumo, ALN-GO1)	21 nt ds-siRNA	2nd gen; 2′-F/2′-O-Me; GalNAc-conjugated.	Subcutaneous	Liver	Primary hyperoxaluria type 1	Hydroxyacid oxidase-1 (*HAO1*) mRNA	2020 (EMA), 2020 (FDA)
Inclisiran (Leqvio, ALN-PCSsc)	21 nt ds-siRNA	2nd gen; 2′-F/2′-O-Me; GalNAc-conjugated.	Subcutaneous	Liver	Atherosclerotic cardiovascular disease, elevated cholesterol, homozygous/heterozygous familial hypercholesterolaemia	Proprotein convertase subtilisin/kexin type 9 (*PCSK9*) mRNA	2020 (EMA)
Volanesorsen (Waylivra)	20-mer ASO	2nd gen; 2′-MOE gapme	Subcutaneous	Liver	Familial chylomicronaemia syndrome	Apolipoprotein CIII (*APOC3*) mRNA	2019 (EMA)
Viltolarsen (Viltepso, NS-065, NCNP-01)	21-mer ASO	3rd gen; 2′-MOE PMO	Intravenous	Muscle	Duchenne muscular dystrophy	*DMD* pre-mRNA splicing (exon 53 skipping)	2020 (FDA)
Givosiran (Givlaari)	21 nt ds-siRNA	2nd gen; 2′-F/2′-O-Me; GalNAc-conjugated	Subcutaneous	Liver	Acute hepatic porphyria	Delta aminolevulinic acid synthase 1 (*ALAS1*) mRNA	2020 (EMA), 2019 (FDA)
Golodirsen (Vyondys 53, SRP-4053)	25-mer ASO	3rd gen; 2′-MOE PMO	Intravenous	Muscle	Duchenne muscular dystrophy	*DMD* pre-mRNA splicing (exon 53 skipping)	2019 (FDA)
Patisiran (Onpattro)	21 nt ds-siRNA	2nd gen; 2′-F/2′-O-Me; liposomal	Intravenous	Liver	Hereditary transthyretin amyloidosis	Transthyretin (*TTR*) mRNA	2018 (EMA), 2019 (FDA)
Inotersen (Tegsedi, AKCEA-TTR-LRx)	20-mer ASO	2nd gen; 2′-MOE; GalNAc-conjugated	Subcutaneous	Liver	Hereditary transthyretin amyloidosis	Transthyretin (*TTR*) mRNA	2018 (EMA), 2018 (FDA)
Eteplirsen (Exondys 51)	30-mer ASO	3rd gen; 2′-MOE PMO	Intravenous	Muscle	Duchenne muscular dystrophy	Dystrophin (*DMD*) pre-mRNA splicing (exon 51 skipping)	2016 (FDA)
Nusinersen (Spinraza, ASO-10-27)	18-mer ASO	2nd gen; 2′-MOE	Intrathecal	Central nervous system	Spinal muscular atrophy	Survival of motor neuron 2 (*SMN2*) pre-mRNA splicing (exon 7 inclusion)	2017 (EMA), 2016 (FDA)
Mipomersen (Kynamro)	20-mer ASO	2nd gen; 2′-MOE gapmer	Subcutaneous	Liver	Homozygous familial hypercholesterolaemia	Apolipoprotein B mRNA	2012 (EMA), 2013 (FDA)
Fomivirsen (Vitravene)	21-mer ASO	1st gen; PT	Intravitreal	Eye	Cytomegalovirus (CMV) retinitis in immunocompromised patients	CMV IE-2 mRNA	1998 (FDA), 1999 (EMA) *

This document lists RNA therapeutics that have been approved by the FDA and/or the European Medicines Agency. Drugs are listed from the most recent approval. ASO, antisense oligonucleotide; ds, double-stranded; GalNAc, *N*-acetylgalactosamine; gen, generation; PMO, phosphoroamidate morpholino oligomer; PT, phosphothiorate; siRNA, small interfering RNA. * Due to the development of effective antiretroviral therapies, marketing was discontinued in 2002.

**Table 2:** Clinical development of RNA therapeutics that have been discontinued.

λTreatment	Type	Amendment & Product Delivery	Mode of Delivery	Destination Site	Disease	Target Gene and Route	Reason for Leaving the Company
Aprinocarsen (ISIS 3521, LY900003)	ASO	1st gen; PT	Intravenous	Tumor	Non-small cell lung cancer	Protein kinase Cα mRNA	No clinical efficacy improvement
ISIS 5132 (CGP 69846 A)	ASO	1st gen; PT	Intravenous	Tumor	Breast cancer, ovarian cancer	*Raf* mRNA	No clinical efficacy improvement
ISIS 104838	ASO	2nd gen; 2′-MOE gapmer	Oral	Joints	Rheumatoid arthritis	TNF mRNA	Company decision related to cost and competition.
PF-4523655 (PF-655)	siRNA	2nd gen; liposomal	Intravitreal	Eye	Age-related macular degeneration, diabetic macular edema	DNA damage-inducible transcript 4 (*DDIT*4) mRNA	No clinical efficacy improvement compared to the current standard of care.
ISIS 329993 (ISIS-CRPRx)	ASO	2nd gen; 2′-MOE	Subcutaneous or intraperitoneal	Heart or joints	Paroxysmal atrial fibrillation, rheumatoid arthritis	C-reactive protein (*CRP*) mRNA	Although it reduced CRP mRNA, clinical efficacy was lacking.
AEG35156 (AEG 161, GEM 640)	ASO	Mixed backbone oligonucleotides	Intravenous	Tumor	Various malignancies	X-linked inhibitor of apoptosis (*XIAP*) mRNA	It lacks clinical efficacy. Increased incidence of chemotherapy-induced peripheral neuropathy.
Custirsen (ISIS 112989, OGX-011, TV-1011)	ASO	2nd gen; 2′-MOE gapmer	Intravenous	Tumor	Prostate cancer, breast cancer	Clusterin (*CLU*) mRNA	Primary end points were not met in phase III trials, indicating a lack of clinical efficacy.
Bevasiranib (Cand5)	siRNA	1st gen; PT	Intravitreal	Eye	Age-related macular degeneration, diabetic macular edema	Vascular endothelial growth factor (*VEGF*) mRNA	The therapeutic effect of TLR3 stimulation, which is independent of sequence, has not been clinically effective.
Oblimersen sodium (G3139, Genasense)	ASO	1st gen; PT	Subcutaneous	Tumor	Various malignancies	*BCL2* mRNA	There was a lack of clinical efficacy due to insufficient delivery, resulting in primary end points not being met.
AGN 211745 (AGN-745, siRNA-027)	siRNA	Chemical composition unclear; carrier-free	Intravitreal	Eye	Age-related macular degeneration, choroidal neovascularization	Vascular endothelial growth factor receptor 1 (*VEGFR1*) mRNA	The therapeutic effect of TLR3 stimulation, which is independent of sequence, has not been clinically effective.
PRO-040201 (TKM-ApoB, ApoB SNALP)	siRNA	Liposomal (stable nucleic acid lipid particle)	Intravenous	Liver	Hypercholesterolaemia	Apolipoprotein B (*APOB*) mRNA	Possible to stimulate the immune system, which may cause flu-like symptoms.
MRX34	miRNA mimic	Liposomal	Intravenous or intratumor	Intravenous or intratumor	Primary liver cancer, advanced or metastatic cancer with or without liver involvement, hematological malignancies	miR-34a targetome	Immune-related adverse events
RG-101	AntimiR	GalNAc-conjugated	Subcutaneous	Liver	Hepatitis C infection	miR-122	Immune-related adverse events
χCobomarsen (MRG-106)	AntimiR	3rd gen; LNA	Subcutaneous or intravenous	Blood or lymphoid organs	Various lymphomas	miR-155	Company decision unrelated to safety or efficacy
χSuvodirsen (WVE-210201)	ASO	1st gen; PT, stereopure	Intravenous	Muscle	Duchenne muscular dystrophy	Dystrophin (*DMD*) pre-mRNA splicing (exon 51 skipping)	The treatment did not show clinical efficacy and did not increase dystrophin levels.
χAganirsen (GS-101)	ASO	1st gen; PT	Topical	Eye	Ischemic central retinal vein occlusion, neovascular glaucoma	Insulin receptor substrate 1 (*IRS1*) mRNA	Problems related to the stability of the formulation
χDCR-PH1	siRNA	Liposomal	Intravenous	Liver	Primary hyperoxaluria type 1 (PH1)	Lactate dehydrogenase A (*LDHA*) mRNA	The focus of development has been on the GalNAc conjugation variant, DCR-PHXC.
χDCR-MYC (DCR-M1711)	siRNA	Liposomal	Intravenous	Tumor	Advanced solid tumors, multiple myeloma, lymphoma	*MYC* mRNA	Despite reducing MYC, there is a lack of clinical efficacy.

Discontinued medications are reported. Abbreviations used in this text include the following: AntimiR, anti-microRNA; ASO, antisense oligonucleotide; GalNac, N-acetylgalactosamine; gen, generation; LNA, locked nucleic acid; PT, phosphothiorate; siRNA, small interfering RNA; and TLR3, Toll-like receptor 3. Please see the related links for more information λ;. λ Related links; Dicerna prioritizes resources to advance GalXc Product candidates: https://www.itnonline.com/content/dicerna-prioritizes-resources-advance-galxc-product-candidates; miRagen decides to discontinue further internal development of cobomarsen: http://investors.miragen.com/press-releases/press-release/2020/miRagen-Announces-Internal-Review-of-Preliminary-Topline-Data-for-the-Phase-2-SOLAR-Clinical-Trial-of-Cobomarsen-in-Patients-with-Cutaneous-T-Cell-Lymphoma-CTCL/default.aspx; PrO-040201: https://www.creative-biolabs.com/gene-therapy/pro040201.htm; Regulus to discontinue clinical development of Hcv candidate rG-101: https://www.pharmaceutical-business-review.com/clinical-trials/news/regulus-to-terminate-development-of-hcv-candidate-rg-101-130617-5841251; Trial termination Aganirsen: http://strong-nvg.com/trial-termination/; Wave Life Sciences discontinues development of suvodirsen for DMD: https://musculardystrophynews.com/2019/12/17/wave-life-sciences-discontinues-suvodirsen-development-for-dmd/, all links accessed on 11 March 2024.

**Table 3:** RNA therapies in phase II or III clinical development.

Treatment	Type	Amendment and Product Delivery	Mode of Delivery	Destination Site	Disease	Target Gene and Route	Phase and Identifier
RG-125 (AZD4076)	Anti-miR-103/107	GalNAc-conjugated antagomiR	Subcutaneous	Liver	Type II diabetes, nonalcoholic fatty liver disease.	miR-103/107	I/II NCT04120493
Prexigebersen (BP1001-A)	ASO	Liposomal	Intravenous	Blood and/or immune cells	Acute myeloid leukemia, chronic myeloid leukemia	*GRB2* mRNA	II NCT01159028; NCT04196257; NCT02781883
WVE-120102	ASO (allele-selective)	Stereopure ASO	Intrathecal	Brain	Huntington disease	U-variant of SNP rs362331 (SNP2) in *HTT* miRNA	I/II NCT03225846, NCT04617860
siG12D-LODER	siRNA	Biodegradable polymeric matrix (PLGA)	Intratumoral	Tumor	Advanced pancreatic cancer	G12D-mutated *KRAS* miRNA	II NCT01188785; NCT01676259
rAAV5-miHTT (AMT-130)	Pri-miR-451 backbone	Adeno-associated viral vector (AAV5)	Intrastriatal	Brain	Huntington disease	Huntingtin (*HTT*) miRNA	I/II NCT04120493
Remlarsen (MRG-201)	miR-29 mimic.	Cholesterol-conjugated.	Intradermal	Skin	Keloid (pathological fibrosis)	miR-29 targetome	II NCT02603224, NCT03601052
Miravirsen (SPC3649)	Anti-miR-122	PS-β-d-oxy-LNA gapmer ODN	Subcutaneous	Liver	Hepatitis C virus infection	miR-122	II NCT01646489, NCT01727934, NCT01872936, NCT01200420
Olpasiran (AMG 890, ARO-LPA	siRNA	GalNAc-conjugated.	Subcutaneous	Liver	Cardiovascular disease	Apolipoprotein A (*LPA*) miRNA	II NCT03626662, NCT04270760
Vupanorsen (AKCEA-ANGPTL3-LRx)	ASO	GalNAc-conjugated.	Subcutaneous	Liver	Dyslipidaemias, hyperlipidaemias, hyperlipoproteinaemias	Angiopoietin-like 3 (*ANGPTL*3) mRNA	II NCT04459767, NCT03371355, NCT04516291
Danvatirsen (IONIS-STAT3-2.5Rx, AZD9150	ASO	GalNAc-conjugated.	Intravenous	Tumor	Metastatic NSCLC, resectable early-stage NSCLC, pancreatic cancer, mismatch repair-deficient colorectal cancer	*STAT3* miRNA	II NCT03819465, NCT03794544, NCT0298357
Cemdisiran (ALN-CC5)	siRNA	GalNAc-conjugated.	Subcutaneous	Blood	Paroxysmal nocturnal hemoglobinuria, IgA nephropathy, Berger disease, glomerulonephritis	Complement 5 miRNA.	II NCT04601844, NCT02352493, NCT03841448, NCT03999840
BMT 101 (cp-asiRNA)	Cell-penetrating asymmetrical siRNA	Carrier-free	Intradermal	Skin	Hypertrophic scar	Connective tissue growth factor (*CTGF*) miRNA	II NCT03133130, NCT04012099
Apatorsen (OGX-427)	ASO	2′-O-MOE-PTO gapmer	Intravenous	Tumor	Squamous cell lung cancer, non-squamous NSCLC, urological neoplasms, metastatic bladder cancer, urinary tract neoplasms, castration-resistant prostate cancer	*HSP27* miRNA	II NCT01120470, NCT01454089, NCT01829113, NCT02423590
Bamosiran (SYL040012)	siRNA	Carrier-free	Topical	Eye	Ocular hypertension, glaucoma	β-Adrenergic receptor 2 (*ADRB2*) miRNA	II NCT00990743, NCT01227291, NCT01739244, NCT02250612
Donidalorsen (IONIS-PKK-LRx, ISIS 721744)	ASO	GalNAc-conjugated PS-2′-MOE ODN	Subcutaneous	Liver	Hereditary angioedema, COVID-19	Prekallikrein (*PKK*) miRNA	II NCT03263507, NCT04030598, NCT04307381, NCT0454992
Sepofarsen (QR-110)	ASO	Chemically modified.	Intravitreal	Eye	Leber congenital amaurosis type 10 (LCA10) is a hereditary or congenital eye disease that can cause blindness and vision and sensation disorders. It may also present with neurological manifestations. LCA10 falls under the category of eye diseases.	c.2991 + 1655A > G-mutated CEP290, pre-miRNA splicing	II/III NCT03140969, NCT03913143, NCT03913130
Tominersen (RO7234292, HTT ASO, IONIS-HTTRx, ISIS-443139, ISIS-HTTRx, RG 6042)	ASO (allele-nonselective)	PS-2′-MOE gapmer	Intrathecal	Brain	Huntington disease	*HTT* miRNA	III NCT02519036, NCT04000594, NCT03342053, NCT03761849, NCT03842969
AKCEA-TTR-LRx	ASO	GalNAc-conjugated.	Subcutaneous	Liver	Hereditary transthyretin-mediated amyloid polyneuropathy	*Transthyretin* (*TTR*) miRNA	III NCT04302064; NCT03728634; NCT04136184; NCT04136171
Alicaforsen (ISIS 2302)	ASO	Phosphorothioate-modified.	Oral	Intestine	Crohn’s disease	*ICAM*1 miRNA	III NCT03473626, NCT00063830, NCT00063414, NCT00048113, NCT02525523
Nedosiran (DCR-PHXC)	siRNA	GalNAc-conjugated.	Subcutaneous	Liver	Primary hyperoxaluria type 1 and type 2 are kidney and urological diseases characterized by excessive oxalate production.	Lactate dehydrogenase A enzyme (*LDHA*) miRNA.	III NCT03392896, NCT04555486, NCT04580420, NCT03847909, NCT04042402
Tivanisiran (SYL1001)	siRNA	Carrier-free	Topical	Eye	Dry eye disease	TRPV1 is a member of the transient receptor potential cation channel subfamily V.	III NCT01438281, NCT01776658, NCT02455999, NCT03108664
Pelacarsen (AKCEA-APO(a)-LRx, TQJ230)	siRNA	GalNAc-conjugated.	Subcutaneous	Liver	Hyperlipoproteinaemia	Apolipoprotein A miRNA	III NCT03070782, NCT03070782, NCT04023552

The table shows the phases of experimentation for miRNA-based therapies and their corresponding levels of study progression. This is indicated by an increase in the study phase. Abbreviations used in this text include the following: ASO, antisense oligonucleotide; GalNAc, *N*-acetylgalactosamine; LNA, locked nucleic acid; LODER, local drug eluter; NSCLC, non-small cell lung cancer; siRNA, small interfering RNA; SNP, single nucleotide polymorphism.

**Table 4:** Clinical trials of miRNAs with therapeutic potential in cardiovascular disease.

Active Principle/Therapeutic Drug Name	Indication	Clinical Phase	Study No./Status	Preclinica/Clinical Study Outcomes	Corporate Sponsor	Related Cardiovascular Studies
miR-132-3p inhibitor (CDR132L)	Stable heart failure	Phase I	NCT04045405 (completed)	[28,170]	Cardior Pharmaceuticals	[28,31,170,171]
miR-122-5p inhibitor (miravirsen)	HCV	Phase I Phase I Phase I Phase IIa	NCT00688012 (completed), NCT00979927 (completed), NCT01646489 (completed), NCT01200420 EudraCT 2010-019057-17 (completed)	[160,172,173]	Santaris Pharma	[160,172,173]
miR-103/107-3p inhibitor (AZD4076)	T2D with NAFLD T2D with NASH	Phase I/IIa Phase I	NCT02826525 (halted for strategic reasons) NCT02612662 (halted for strategic reasons)		AstraZeneca	[174]
miR-122-5p inhibitor (RG-101)	HCV	Phase II PhaseII PhaseIIb Phase IIb	EudraCT 2015-004702-42 (completed), EudraCT 2015-001535-21 (completed), EudraCT 2013-002978-49 (completed), EudraCT 2016-002069-77 (completed)	[175,176]	Regulus Therapeutics	
miR-16-5p mimic (TargomiR)	Malignant pleural mesothelioma	Phase I	NCT02369198 (completed)	[177]	Asbestos Diseases Research Foundation	
miR-17-5p inhibitor (RGLS4326)	ADPKD	Phase Ib	NCT04536688 (completed)	[178]	Regulus Therapeutics	[179]
miR-155-5p inhibitor cobomarsen (MRG-106)	Cutaneous T-cell lymphoma	Phase I Phase II	NCT02580552 (completed) NCT03713320 (terminated for strategic reasons	[180]	miRagen Therapeutics (now Viridian Therapeutic)	[32,34]
miR-92a-3p inhibitor (MRG-110)	Wound healing	Phase I Phase I Phase I	NCT03603431 (completed) NCT03494712 (completed) EUDRA-CT 2017-004180-12 (completed)	[181]	miRagen Therapeutics (now Viridian Therapeutic)	[45,85,134]
miR-21-5p inhibitor lademirsen (RG-012)	Alport’s syndrome	Phase I Phase II	NCT02603224 (completed) NCT02855268 (ongoing)		Genzyme/Sanofi	
miR-29-3p mimic remlarsen (MRG-201)	Keloid scar formation	Phase I Phase II	NCT02603224 (completed) NCT03601052 (completed)		miRagen Therapeutics (now Viridian Therapeutic)	[17,22,23,173]
miR-34a-5p mimic (MRX-34)	Advanced cancer	Phase I	NCT01829971 (terminated due to serious adverse effects)	[128]	Mirna Therapeutics	[177,182]

The table summarizes 19 clinical trials that have used microRNA-based therapeutics. Abbreviations used in this text include the following: ADPKD, autosomal dominant polycystic kidney disease; T2D, type 2 diabetes; NAFLD, nonalcoholic fatty liver disease; NASH, nonalcoholic steatohepatitis; HCV, hepatitis C virus. Refs. [17,22,23,28,32,34,85,160,170,171,172,173,174,175,176,177,178,179,180,181,182].

## References

[1] Wightman B., Ha I., Ruvkun G. (1993). Posttranscriptional regulation of the heterochronic gene lin-14 by lin-4 mediates temporal pattern formation in C. elegans. Cell.

[2] Huang W., Paul D., Calin G.A., Bayraktar R. (2023). miR-142: A Master Regulator in Hematological Malignancies and Therapeutic Opportunities. Cells.

[3] Lee R.C., Feinbaum R.L., Ambros V. (1993). The C. elegans heterochronic gene lin-4 encodes small RNAs with antisense complementarity to lin-14. Cell.

[4] Jan C.H., Friedman R.C., Ruby J.G., Bartel D.P. (2011). Formation, regulation and evolution of Caenorhabditis elegans 3′UTRs. Nature.

[5] Friedman R.C., Farh K.K.-H., Burge C.B., Bartel D.P. (2009). Most mammalian mRNAs are conserved targets of microRNAs. Genome Res..

[6] Ha I., Wightman B., Ruvkun G. (1996). A bulged lin-4/lin-14 RNA duplex is sufficient for Caenorhabditis elegans lin-14 temporal gradient formation. Genes Dev..

[7] Grimson A., Farh K.K.-H., Johnston W.K., Garrett-Engele P., Lim L.P., Bartel D.P. (2007). MicroRNA Targeting Specificity in Mammals: Determinants beyond Seed Pairing. Mol. Cell.

[8] Kozomara A., Birgaoanu M., Griffiths-Jones S. (2019). miRBase: From microRNA sequences to function. Nucleic Acids Res..

[9] Fromm B., Domanska D., Høye E., Ovchinnikov V., Kang W., Aparicio-Puerta E., Johansen M., Flatmark K., Mathelier A., Hovig E. (2020). MirGeneDB 2.0: The metazoan microRNA complement. Nucleic Acids Res..

[10] Kim K., Baek S.C., Lee Y.-Y., Bastiaanssen C., Kim J., Kim H., Kim V.N. (2021). A quantitative map of human primary microRNA processing sites. Mol. Cell.

[11] Nappi F., Alzamil A., Singh S.S.A., Spadaccio C., Bonnet N. (2023). Current Knowledge on the Interaction of Human Cytomegalovirus Infection, Encoded miRNAs, and Acute Aortic Syndrome. Viruses.

[12] Nappi F., Singh S.S.A., Jitendra V., Alzamil A., Schoell T. (2023). The Roles of microRNAs in the Cardiovascular System. Int. J. Mol. Sci..

[13] Matsui M., Prakash T.P., Corey D.R. (2016). Argonaute 2-dependent Regulation of Gene Expression by Single-stranded miRNA Mimics. Mol. Ther..

[14] Eulalio A., Mano M., Ferro M.D., Zentilin L., Sinagra G., Zacchigna S., Giacca M. (2012). Functional screening identifies miRNAs inducing cardiac regeneration. Nature.

[15] Hinkel R., Ramanujam D., Kaczmarek V., Howe A., Klett K., Beck C., Dueck A., Thum T., Laugwitz K.-L., Maegdefessel L. (2020). AntimiR-21 Prevents Myocardial Dysfunction in a Pig Model of Ischemia/Reperfusion Injury. J. Am. Coll. Cardiol..

[16] Ganesan J., Ramanujam D., Sassi Y., Ahles A., Jentzsch C., Werfel S., Leierseder S., Loyer X., Giacca M., Zentilin L. (2013). MiR-378 Controls Cardiac Hypertrophy by Combined Repression of Mitogen-Activated Protein Kinase Pathway Factors. Circulation.

[17] Sassi Y., Avramopoulos P., Ramanujam D.P., Grüter L., Werfel S., Giosele S., Brunner A.-D., Esfandyari D., Papadopoulou A.-S., De Strooper B. (2017). Cardiac myocyte miR-29 promotes pathological remodeling of the heart by activating Wnt signaling. Nat. Commun..

[18] Thum T., Gross C., Fiedler J., Fischer T., Kissler S., Bussen M., Galuppo P., Just S., Rottbauer W., Frantz S. (2008). MicroRNA-21 contributes to myocardial disease by stimulating MAP kinase signalling in fibroblasts. Nature.

[19] Ji R., Cheng Y., Yue J., Yang J., Liu X., Chen H., Dean D.B., Zhang C. (2007). microrna expression signature and antisense-mediated depletion reveal an essential role of microrna in vascular neointimal lesion formation. Circ. Res..

[20] Ramanujam D., Sassi Y., Laggerbauer B., Engelhardt S. (2016). Viral Vector-Based Targeting of miR-21 in Cardiac Nonmyocyte Cells Reduces Pathologic Remodeling of the Heart. Mol. Ther..

[21] Van Rooij E., Sutherland L.B., Thatcher J.E., DiMaio J.M., Naseem R.H., Marshall W.S., Hill J.A., Olson E.N. (2008). Dysregulation of microRNAs after myocardial infarction reveals a role of miR-29 in cardiac fibrosis. Proc. Natl. Acad. Sci. USA.

[22] Boon R.A., Seeger T., Heydt S., Fischer A., Hergenreider E., Horrevoets A.J., Vinciguerra M., Rosenthal N., Sciacca S., Pilato M. (2011). MicroRNA-29 in Aortic Dilation: Implications for Aneurysm Formation. Circ. Res..

[23] Maegdefessel L., Azuma J., Toh R., Merk D.R., Deng A., Chin J.T., Raaz U., Schoelmerich A.M., Raiesdana A., Leeper N.J. (2012). Inhibition of microRNA-29b reduces murine abdominal aortic aneurysm development. J. Clin. Investig..

[24] McDonald R.A., White K.M., Wu J., Cooley B.C., Robertson K.E., Halliday C.A., McClure J.D., Francis S., Lu R., Kennedy S. (2013). miRNA-21 is dysregulated in response to vein grafting in multiple models and genetic ablation in mice attenuates neointima formation. Eur. Heart J..

[25] Eken S.M., Christersdottir T., Winski G., Sangsuwan T., Jin H., Chernogubova E., Pirault J., Sun C., Simon N., Winter H. (2019). miR-29b Mediates the Chronic Inflammatory Response in Radiotherapy-Induced Vascular Disease. JACC Basic Transl. Sci..

[26] Zeng Z., Xia L., Fan X., Ostriker A.C., Yarovinsky T., Su M., Zhang Y., Peng X., Xie Y., Pi L. (2019). Platelet-derived miR-223 promotes a phenotypic switch in arterial injury repair. J. Clin. Investig..

[27] Hinkel R., Penzkofer D., Zühlke S., Fischer A., Husada W., Xu Q.F., Baloch E., van Rooij E., Zeiher A.M., Kupatt C. (2013). Inhibition of microRNA-92a protects against ischemia/reperfusion injury in a large-animal model. Circulation.

[28] Foinquinos A., Batkai S., Genschel C., Viereck J., Rump S., Gyöngyösi M., Traxler D., Riesenhuber M., Spannbauer A., Lukovic D. (2020). Preclinical development of a miR-132 inhibitor for heart failure treatment. Nat. Commun..

[29] Corsten M.F., Papageorgiou A., Verhesen W., Carai P., Lindow M., Obad S., Summer G., Coort S.L.M., Hazebroek M., Van Leeuwen R. (2012). MicroRNA Profiling Identifies MicroRNA-155 as an Adverse Mediator of Cardiac Injury and Dysfunction During Acute Viral Myocarditis. Circ. Res..

[30] Bonauer A., Carmona G., Iwasaki M., Mione M., Koyanagi M., Fischer A., Burchfield J., Fox H., Doebele C., Ohtani K. (2009). MicroRNA-92a Controls Angiogenesis and Functional Recovery of Ischemic Tissues in Mice. Science.

[31] Ucar A., Gupta S.K., Fiedler J., Erikci E., Kardasinski M., Batkai S., Dangwal S., Kumarswamy R., Bang C., Holzmann A. (2012). The miRNA-212/132 family regulates both cardiac hypertrophy and cardiomyocyte autophagy. Nat. Commun..

[32] Heymans S., Corsten M.F., Verhesen W., Carai P., van Leeuwen R.E., Custers K., Peters T., Hazebroek M., Stöger L., Wijnands E. (2013). Macrophage MicroRNA-155 Promotes Cardiac Hypertrophy and Failure. Circulation.

[33] Lu Y., Zhang Y., Wang N., Pan Z., Gao X., Zhang F., Zhang Y., Shan H., Luo X., Bai Y. (2010). MicroRNA-328 Contributes to Adverse Electrical Remodeling in Atrial Fibrillation. Circulation.

[34] Pankratz F., Bemtgen X., Zeiser R., Leonhardt F., Kreuzaler S., Hilgendorf I., Smolka C., Helbing T., Hoefer I., Esser J.S. (2015). MicroRNA-155 Exerts Cell-Specific Antiangiogenic but Proarteriogenic Effects During Adaptive Neovascularization. Circulation.

[35] Halkein J., Tabruyn S.P., Ricke-Hoch M., Haghikia A., Nguyen N.-Q., Scherr M., Castermans K., Malvaux L., Lambert V., Thiry M. (2013). MicroRNA-146a is a therapeutic target and biomarker for peripartum cardiomyopathy. J. Clin. Investig..

[36] Sun X., Icli B., Wara A.K., Belkin N., He S., Kobzik L., Hunninghake G.M., Vera M.P., Blackwell T.S., MICU Registry (2012). MicroRNA-181b regulates NF-κB–mediated vascular inflammation. J. Clin. Investig..

[37] da Costa Martins P.A., Salic K., Gladka M.M., Armand A.-S., Leptidis S., El Azzouzit H., Hansen A., Coenen-de Roo C.J., Bierhuizen M.F., Van Der Nagel R. (2010). MicroRNA-199b targets the nuclear kinase Dyrk1a in an auto-amplification loop promoting calcineurin/NFAT signalling. Nat. Cell Biol..

[38] Duygu B., Poels E.M., Juni R., Bitsch N., Ottaviani L., Olieslagers S., de Windt L.J., da Costa Martins P.A. (2017). miR-199b-5p is a regulator of left ventricular remodeling following myocardial infarction. Non-Coding RNA Res..

[39] van Rooij E., Sutherland L.B., Liu N., Williams A.H., McAnally J., Gerard R.D., Richardson J.A., Olson E.N. (2006). A signature pattern of stress-responsive microRNAs that can evoke cardiac hypertrophy and heart failure. Proc. Natl. Acad. Sci. USA.

[40] Aurora A.B., Mahmoud A.I., Luo X., Johnson B.A., van Rooij E., Matsuzaki S., Humphries K.M., Hill J.A., Bassel-Duby R., Sadek H.A. (2012). MicroRNA-214 protects the mouse heart from ischemic injury by controlling Ca^2+^ overload and cell death. J. Clin. Investig..

[41] Liu X., Xiao J., Zhu H., Wei X., Platt C., Damilano F., Xiao C., Bezzerides V., Boström P., Che L. (2015). miR-222 Is Necessary for Exercise-Induced Cardiac Growth and Protects against Pathological Cardiac Remodeling. Cell Metab..

[42] Verjans R., Peters T., Beaumont F.J., van Leeuwen R., van Herwaarden T., Verhesen W., Munts C., Bijnen M., Henkens M., Diez J. (2018). MicroRNA-221/222 family counteracts myocardial fibrosis in pressure overload-induced heart failure. Hypertension.

[43] Carè A., Catalucci D., Felicetti F., Bonci D., Addario A., Gallo P., Bang M.L., Segnalini P., Gu Y., Dalton N.D. (2007). Mi-croRNA-133 controls cardiac hypertrophy. Nat. Med..

[44] Besser J., Malan D., Wystub K., Bachmann A., Wietelmann A., Sasse P., Fleischmann B.K., Braun T., Boettger T. (2014). MiR-NA-1/133a clusters regulate adrenergic control of cardiac repolarization. PLoS ONE.

[45] Porrello E.R., Mahmoud A.I., Simpson E., Johnson B.A., Grinsfelder D., Canseco D., Mammen P.P., Rothermel B.A., Olson E.N., Sadek H.A. (2013). Regulation of neonatal and adult mammalian heart regeneration by the miR-15 family. Proc. Natl. Acad. Sci. USA.

[46] Fiedler J., Stöhr A., Gupta S.K., Hartmann D., Holzmann A., Just A., Hansen A., Hilfiker-Kleiner D., Eschenhagen T., Thum T. (2014). Functional microRNA library screening identifies the hypoxamir miR-24 as a potent regulator of smooth muscle cell prolif-eration and vascularization. Antioxid. Redox Signal..

[47] Meloni M., Marchetti M., Garner K., Littlejohns B., Sala-Newby G., Xenophontos N., Floris I., Suleiman M.-S., Madeddu P., Caporali A. (2013). Local Inhibition of MicroRNA-24 Improves Reparative Angiogenesis and Left Ventricle Remodeling and Function in Mice With Myocardial Infarction. Mol. Ther..

[48] Boon R.A., Iekushi K., Lechner S., Seeger T., Fischer A., Heydt S., Kaluza D., Tréguer K., Carmona G., Bonauer A. (2013). MicroRNA-34a regulates cardiac ageing and function. Nature.

[49] Badi I., Mancinelli L., Polizzotto A., Ferri D., Zeni F., Burba I., Milano G., Brambilla F., Saccu C., Bianchi M.E. (2018). miR-34a Promotes Vascular Smooth Muscle Cell Calcification by Downregulating SIRT1 (Sirtuin 1) and Axl (AXL Receptor Tyrosine Kinase). Arterioscler. Thromb. Vasc. Biol..

[50] Laggerbauer B., Engelhardt S. (2022). MicroRNAs as therapeutic targets in cardiovascular disease. J. Clin. Investig..

[51] Treiber T., Treiber N., Meister G. (2019). Regulation of microRNA biogenesis and its crosstalk with other cellular pathways. Nat. Rev. Mol. Cell Biol..

[52] Bartel D.P. (2018). Metazoan microRNAs. Cell.

[53] Khvorova A., Reynolds A., Jayasena S.D. (2003). Functional siRNAs and miRNAs Exhibit Strand Bias. Cell.

[54] Schwarz D.S., Hutvágner G., Du T., Xu Z., Aronin N., Zamore P.D. (2003). Asymmetry in the Assembly of the RNAi Enzyme Complex. Cell.

[55] Bang C., Batkai S., Dangwal S., Gupta S.K., Foinquinos A., Holzmann A., Just A., Remke J., Zimmer K., Zeug A. (2014). Cardiac fibroblast–derived microRNA passenger strand-enriched exosomes mediate cardiomyocyte hypertrophy. J. Clin. Investig..

[56] Santovito D., Egea V., Bidzhekov K., Natarelli L., Mourão A., Blanchet X., Wichapong K., Aslani M., Brunßen C., Horckmans M. (2020). Noncanonical inhibition of caspase-3 by a nuclear microRNA confers endothelial protection by autophagy in athero-sclerosis. Sci. Transl. Med..

[57] Li H., Zhan J., Zhao Y., Fan J., Yuan S., Yin Z., Dai B., Chen C., Wang D.W. (2019). Identification of ncRNA-Mediated Functions of Nucleus-Localized miR-320 in Cardiomyocytes. Mol. Ther. Nucleic Acids.

[58] Agarwal V., Bell G.W., Nam J.-W., Bartel D.P. (2015). Predicting effective microRNA target sites in mammalian mRNAs. eLife.

[59] Broughton J.P., Lovci M.T., Huang J.L., Yeo G.W., Pasquinelli A.E. (2016). Pairing beyond the Seed Supports MicroRNA Targeting Specificity. Mol. Cell.

[60] Seth P.P., Tanowitz M., Bennett C.F. (2019). Selective tissue targeting of synthetic nucleic acid drugs. J. Clin. Investig..

[61] Gebert L.F.R., Macrae I.J. (2019). Regulation of microRNA function in animals. Nat. Rev. Mol. Cell Biol..

[62] Yang A., Bofill-De Ros X., Shao T.-J., Jiang M., Li K., Villanueva P., Dai L., Gu S. (2019). 3′ Uridylation Confers miRNAs with Non-canonical Target Repertoires. Mol. Cell.

[63] Van der Kwast R.V., Parma L., van der Bent M.L., van Ingen E., Baganha F., Peters H.A., Goossens E.A., Simons K.H., Palmen M., de Vries M.R. (2020). Adenosine-to-Inosine Editing of Vasoactive MicroRNAs Alters Their Targetome and Function in Ischemia. Mol. Ther. Nucleic Acids.

[64] McGahon M.K., Yarham J.M., Daly A., Guduric-Fuchs J., Ferguson L.J., Simpson D.A., Collins A. (2013). Distinctive Profile of IsomiR Expression and Novel MicroRNAs in Rat Heart Left Ventricle. PLoS ONE.

[65] van der Kwast R.V., van Ingen E., Parma L., Peters H.A., Quax P.H., Nossent A.Y. (2018). Adenosine-to-Inosine Editing of MicroRNA-487b Alters Target Gene Selection After Ischemia and Promotes Neovascularization. Circ. Res..

[66] Van der Kwast R.V.C.T., Woudenberg T., Quax P.H.A., Nossent A.Y. (2020). MicroRNA-411 and Its 5′-IsomiR have distinct targets and functions and are differentially regulated in the vasculature under ischemia. Mol. Ther..

[67] Kingston E.R., Bartel D.P. (2019). Global analyses of the dynamics of mammalian microRNA metabolism. Genome Res..

[68] Marzi M.J., Ghini F., Cerruti B., de Pretis S., Bonetti P., Giacomelli C., Gorski M.M., Kress T., Pelizzola M., Muller H. (2016). Degradation dynamics of microRNAs revealed by a novel pulse-chase approach. Genome Res..

[69] Han J., LaVigne C.A., Jones B.T., Zhang H., Gillett F., Mendell J.T. (2020). A ubiquitin ligase mediates target-directed microRNA decay independently of tailing and trimming. Science.

[70] Shi C.Y., Kingston E.R., Kleaveland B., Lin D.H., Stubna M.W., Bartel D.P. (2020). The ZSWIM8 ubiquitin ligase mediates tar-get-directed microRNA degradation. Science.

[71] Bitetti A., Mallory A.C., Golini E., Carrieri C., Gutiérrez H.C., Perlas E., Pérez-Rico Y.A., Tocchini-Valentini G.P., Enright A.J., Norton W.H.J. (2018). MicroRNA degradation by a conserved target RNA regulates animal behavior. Nat. Struct. Mol. Biol..

[72] Cabili M.N., Trapnell C., Goff L., Koziol M., Tazon-Vega B., Regev A., Rinn J.L. (2011). Integrative annotation of human large intergenic noncoding RNAs reveals global properties and specific subclasses. Genes Dev..

[73] Fabbri M., Girnita L., Varani G., Calin G.A. (2019). Decrypting noncoding RNA interactions, structures, and functional networks. Genome Res..

[74] Rinn J.L., Chang H.Y. (2012). Genome Regulation by Long Noncoding RNAs. Annu. Rev. Biochem..

[75] Guttman M., Rinn J.L. (2012). Modular regulatory principles of large non-coding RNAs. Nature.

[76] Dragomir M., Calin G.A. (2018). Circular RNAs in Cancer—Lessons Learned From microRNAs. Front. Oncol..

[77] Hansen T.B., Jensen T.I., Clausen B.H., Bramsen J.B., Finsen B., Damgaard C.K., Kjems J. (2013). Natural RNA circles function as efficient microRNA sponges. Nature.

[78] He J.H., Li Y.G., Han Z.P., Zhou J.B., Chen W.M., Lv Y.B., He M.L., Zuo J.D., Zheng L. (2018). The CircRNA-ACAP2/Hsa-miR-21-5p/ Tiam1 Regulatory Feedback Circuit Affects the Proliferation, Migration, and Invasion of Colon Cancer SW480 Cells. Cell Physiol. Biochem..

[79] Wang K., Jiang Z., Webster K.A., Chen J., Hu H., Zhou Y., Zhao J., Wang L., Wang Y., Zhong Z. (2017). Enhanced cardio-protection by human endometrium mesenchymal stem cells driven by exosomal microRNA-21. Stem Cells Transl. Med..

[80] Cheng M., Yang J., Zhao X., Zhang E., Zeng Q., Yu Y., Yang L., Wu B., Yi G., Mao X. (2019). Circulating myocardial mi-cro-RNAs from infarcted hearts are carried in exosomes and mobilise bone marrow progenitor cells. Nat. Commun..

[81] Zheng D., Huo M., Li B., Wang W., Piao H., Wang Y., Zhu Z., Li D., Wang T., Liu K. (2021). The Role of Exosomes and Exosomal MicroRNA in Cardiovascular Disease. Front. Cell Dev. Biol..

[82] Kesidou D., da Costa Martins P.A., De Windt L.J., Brittan M., Beqqali A., Baker A.H. (2020). Extracellular Vesicle miRNAs in the Promotion of Cardiac Neovascularisation. Front. Physiol..

[83] Ottaviani L., Sansonetti M., Martins P.A.d.C. (2018). Myocardial cell-to-cell communication via microRNAs. Non-Coding RNA Res..

[84] Zernecke A., Bidzhekov K., Noels H., Shagdarsuren E., Gan L., Denecke B., Hristov M., Köppel T., Jahantigh M.N., Lutgens E. (2009). Delivery of MicroRNA-126 by Apoptotic Bodies Induces CXCL12-Dependent Vascular Protection. Sci. Signal..

[85] Martello A., Mellis D., Meloni M., Howarth A., Ebner D., Caporali A., Zen A.A.H. (2018). Phenotypic miRNA Screen Identifies miR-26b to Promote the Growth and Survival of Endothelial Cells. Mol. Ther. Nucleic Acids.

[86] Eder A., Vollert I., Hansen A., Eschenhagen T. (2015). Human engineered heart tissue as a model system for drug testing. Adv. Drug Deliv. Rev..

[87] Fischer C., Milting H., Fein E., Reiser E., Lu K., Seidel T., Schinner C., Schwarzmayr T., Schramm R., Tomasi R. (2019). Long-term functional and structural preservation of precision-cut human myocardium under continuous electromechanical stimulation in vitro. Nat. Commun..

[88] Meekel J.P., Groeneveld M.E., Bogunovic N., Keekstra N., Musters R.J.P., Zandieh-Doulabi B., Pals G., Micha D., Niessen H.W.M., Wiersema A.M. (2018). An in vitro method to keep human aortic tissue sections functionally and structurally intact. Sci. Rep..

[89] Thomas R.C., Singh A., Cowley P.M., Myagmar B.-E., Montgomery M.D., Swigart P.M., De Marco T., Baker A.J., Simpson P.C. (2016). A Myocardial Slice Culture Model Reveals Alpha-1A-Adrenergic Receptor Signaling in the Human Heart. JACC Basic Transl. Sci..

[90] Kang C., Qiao Y., Li G., Baechle K., Camelliti P., Rentschler S., Efimov I.R. (2016). Human Organotypic Cultured Cardiac Slices: New Platform For High Throughput Preclinical Human Trials. Sci. Rep..

[91] Esfandyari D., Idrissou B.M.G., Hennis K., Avramopoulos P., Dueck A., El-Battrawy I., Grüter L., Meier M.A., Näger A.C., Ramanujam D. (2022). MicroRNA-365 regulates human cardiac action potential duration. Nat. Commun..

[92] Mariani S.A., Li Z., Rice S., Krieg C., Fragkogianni S., Robinson M., Vink C.S., Pollard J.W., Dzierzak E. (2019). Pro-inflammatory Aorta-Associated Macrophages Are Involved in Embryonic Development of Hematopoietic Stem Cells. Immunity.

[93] Litviňuková M., Talavera-López C., Maatz H., Reichart D., Worth C.L., Lindberg E.L., Kanda M., Polanski K., Heinig M., Lee M. (2020). Cells of the adult human heart. Nature.

[94] Wang L., Yu P., Zhou B., Song J., Li Z., Zhang M., Guo G., Wang Y., Chen X., Han L. (2020). Single-cell reconstruction of the adult human heart during heart failure and recovery reveals the cellular landscape underlying cardiac function. Nat. Cell Biol..

[95] Hücker S.M., Fehlmann T., Werno C., Weidele K., Lüke F., Schlenska-Lange A., Klein C.A., Keller A., Kirsch S. (2021). Single-cell microRNA sequencing method comparison and application to cell lines and circulating lung tumor cells. Nat. Commun..

[96] Mokou M., Klein J., Makridakis M., Bitsika V., Bascands J.-L., Saulnier-Blache J.S., Mullen W., Sacherer M., Zoidakis J., Pieske B. (2019). Proteomics based identification of KDM5 histone demethylases associated with cardiovascular disease. EBioMedicine.

[97] Yin X., Wanga S., Fellows A.L., Barallobre-Barreiro J., Lu R., Davaapil H., Franken R., Fava M., Baig F., Skroblin P. (2019). Glycoproteomic Analysis of the Aortic Extracellular Matrix in Marfan Patients. Arterioscler. Thromb. Vasc. Biol..

[98] Doll S., Dreßen M., Geyer P.E., Itzhak D.N., Braun C., Doppler S.A., Meier F., Deutsch M.-A., Lahm H., Lange R. (2017). Region and cell-type resolved quantitative proteomic map of the human heart. Nat. Commun..

[99] Brunner A., Thielert M., Vasilopoulou C., Ammar C., Coscia F., Mund A., Hoerning O.B., Bache N., Apalategui A., Lubeck M. (2022). Ultra-high sensitivity mass spectrometry quantifies single-cell proteome changes upon perturbation. Mol. Syst. Biol..

[100] McGeary S.E., Lin K.S., Shi C.Y., Pham T.M., Bisaria N., Kelley G.M., Bartel D.P. (2019). The biochemical basis of microRNA targeting efficacy. Science.

[101] Werfel S., Leierseder S., Ruprecht B., Kuster B., Engelhardt S. (2017). Preferential microRNA targeting revealed by in vivo competitive binding and differential Argonaute immunoprecipitation. Nucleic Acids Res..

[102] Bissels U., Wild S., Tomiuk S., Holste A., Hafner M., Tuschl T., Bosio A. (2009). Absolute quantification of microRNAs by using a universal reference. RNA.

[103] Denzler R., Agarwal V., Stefano J., Bartel D.P., Stoffel M. (2014). Assessing the ceRNA Hypothesis with Quantitative Measurements of miRNA and Target Abundance. Mol. Cell.

[104] Bosson A.D., Zamudio J.R., Sharp P.A. (2014). Endogenous miRNA and Target Concentrations Determine Susceptibility to Potential ceRNA Competition. Mol. Cell.

[105] Denzler R., McGeary S.E., Title A.C., Agarwal V., Bartel D.P., Stoffel M. (2016). Impact of MicroRNA Levels, Target-Site Complementarity, and Cooperativity on Competing Endogenous RNA-Regulated Gene Expression. Mol. Cell.

[106] Calin G.A., Croce C.M. (2006). MicroRNA-Cancer Connection: The Beginning of a New Tale. Cancer Res..

[107] Lenkala D., LaCroix B., Gamazon E.R., Geeleher P., Im H.K., Huang R.S. (2014). The impact of microRNA expression on cellular proliferation. Hum. Genet..

[108] Ivey K.N., Srivastava D. (2015). MicroRNAs as Developmental Regulators. Cold Spring Harb. Perspect. Biol..

[109] Gutschner T., Diederichs S. (2012). The hallmarks of cancer. RNA Biol..

[110] Schmitt A.M., Chang H.Y. (2016). Long Noncoding RNAs in Cancer Pathways. Cancer Cell.

[111] Mehta A., Baltimore D. (2016). MicroRNAs as regulatory elements in immune system logic. Nat. Rev. Immunol..

[112] Amit M., Takahashi H., Dragomir M.P., Lindemann A., Gleber-Netto F.O., Pickering C.R., Anfossi S., Osman A.A., Cai Y., Wang R. (2020). Loss of p53 drives neuron reprogramming in head and neck cancer. Nature.

[113] Andersen R.E., Lim D.A. (2018). Forging our understanding of lncRNAs in the brain. Cell Tissue Res..

[114] Constantin L. (2018). Circular RNAs and Neuronal Development. Adv. Exp. Med. Biol..

[115] Ling H., Fabbri M., Calin G.A. (2013). MicroRNAs and other non-coding RNAs as targets for anticancer drug development. Nat. Rev. Drug Discov..

[116] Rupaimoole R., Slack F.J. (2017). MicroRNA therapeutics: Towards a new era for the management of cancer and other diseases. Nat. Rev. Drug Discov..

[117] Nappi F., Avtaar Singh S.S., Padala M., Attias D., Nejjari M., Mihos C.G., Benedetto U., Michler R. (2019). The Choice of Treatment in Ischemic Mitral Regurgitation With Reduced Left Ventricular Function. Ann. Thorac. Surg..

[118] Khvorova A., Watts A.K.J.K. (2017). The chemical evolution of oligonucleotide therapies of clinical utility. Nat. Biotechnol..

[119] Ochoa S., Milam V.T. (2020). Modified Nucleic Acids: Expanding the Capabilities of Functional Oligonucleotides. Molecules.

[120] Crooke S.T. (2017). Molecular Mechanisms of Antisense Oligonucleotides. Nucleic Acid Ther..

[121] Singh N.N., Luo D., Singh R.N. (2018). Pre-mRNA Splicing Modulation by Antisense Oligonucleotides. Methods Mol. Biol..

[122] Elbashir S.M., Harborth J., Lendeckel W., Yalcin A., Weber K., Tuschl T. (2001). Duplexes of 21-nucleotide RNAs mediate RNA interference in cultured mammalian cells. Nature.

[123] Rao D.D., Senzer N., Wang Z., Kumar P., Jay C.M., Nemunaitis J. (2013). Bifunctional short hairpin RNA (bi-shRNA): Design and pathway to clinical application. Methods Mol. Biol..

[124] Rao D.D., Jay C., Wang Z., Luo X., Kumar P., Eysenbach H., Ghisoli M., Senzer N., Nemunaitis J. (2016). Preclinical Justification of pbi-shRNA EWS/FLI1 Lipoplex (LPX) Treatment for Ewing’s Sarcoma. Mol. Ther..

[125] Wang Z., Jay C.M., Evans C., Kumar P., Phalon C., Rao D.D., Senzer N., Nemunaitis J. (2016). Preclinical Biodistribution and Safety Evaluation of a pbi-shRNA STMN1 Lipoplex after Subcutaneous Delivery. Toxicol. Sci..

[126] van Rooij E., Kauppinen S. (2014). Development of microRNA therapeutics is coming of age. EMBO Mol. Med..

[127] Beg M.S., Brenner A.J., Sachdev J., Borad M., Kang Y.-K., Stoudemire J., Smith S., Bader A.G., Kim S., Hong D.S. (2016). Phase I study of MRX34, a liposomal miR-34a mimic, administered twice weekly in patients with advanced solid tumors. Investig. New Drugs.

[128] Hong D.S., Kang Y.K., Borad M., Sachdev J., Ejadi S., Lim H.Y., Brenner A.J., Park K., Lee J.L., Kim T.Y. (2020). Phase 1 study of MRX34, a liposomal miR-34a mimic, in patients with advanced solid tumours. Br. J. Cancer.

[129] van Zandwijk N., Pavlakis N., Kao S.C., Linton A., Boyer M.J., Clarke S., Huynh Y., Chrzanowska A., Fulham M.J., Bailey D.L. (2017). Safety and activity of microRNA-loaded minicells in patients with recurrent malignant pleural mesothelioma: A first-in-man, phase 1, open-label, dose-escalation study. Lancet Oncol..

[130] Krützfeldt J., Rajewsky N., Braich R., Rajeev K.G., Tuschl T., Manoharan M., Stoffel M. (2005). Silencing of microRNAs in vivo with ‘antagomirs’. Nature.

[131] Gebert L.F.R., Rebhan M.A.E., Crivelli S.E.M., Denzler R., Stoffel M., Hall J. (2013). Miravirsen (SPC3649) can inhibit the biogenesis of miR-122. Nucleic Acids Res..

[132] Ebert M.S., Neilson J.R., Sharp P.A. (2007). MicroRNA sponges: Competitive inhibitors of small RNAs in mammalian cells. Nat. Methods.

[133] Lima J.F., Cerqueira L., Figueiredo C., Oliveira C., Azevedo N.F. (2018). Anti-miRNA oligonucleotides: A comprehensive guide for design. RNA Biol..

[134] Kluiver J., Slezak-Prochazka I., Smigielska-Czepiel K., Halsema N., Kroesen B.-J., Berg A.v.D. (2012). Generation of miRNA sponge constructs. Methods.

[135] Chang S. (2018). Construction of Multi-Potent MicroRNA Sponge and Its Functional Evaluation. Methods Mol. Biol..

[136] Jung J., Yeom C., Choi Y.-S., Kim S., Lee E., Park M.J., Kang S.W., Kim S.B., Chang S. (2015). Simultaneous inhibition of multiple oncogenic miRNAs by a multi-potent microRNA sponge. Oncotarget.

[137] Das S., Kohr M., Dunkerly-Eyring B., Lee D.I., Bedja D., Kent O.A., Leung A.K.L., Henao-Mejia J., Flavell R.A., Steenbergen C. (2017). Divergent Effects of miR-181 Family Members on Myocardial Function Through Protective Cytosolic and Detrimental Mitochondrial microRNA Targets. J. Am. Heart Assoc..

[138] Bernardo B.C., Gregorevic P., Ritchie R.H., McMullen J.R. (2018). Generation of MicroRNA-34 Sponges and Tough Decoys for the Heart: Developments and Challenges. Front. Pharmacol..

[139] Wang Z. (2011). The principles of MiRNA-masking antisense oligonucleotides technology. Methods Mol. Biol..

[140] Murakami K., Miyagishi M. (2014). Tiny masking locked nucleic acids effectively bind to mRNA and inhibit binding of microRNAs in relation to thermodynamic stability. Biomed. Rep..

[141] Gilot D., Migault M., Bachelot L., Journé F., Rogiers A., Donnou-Fournet E., Mogha A., Mouchet N., Pinel-Marie M.-L., Mari B. (2017). A non-coding function of TYRP1 mRNA promotes melanoma growth. Nat. Cell Biol..

[142] Slack F.J., Chinnaiyan A.M. (2019). The Role of Non-coding RNAs in Oncology. Cell.

[143] Shah M.Y., Ferrajoli A., Sood A.K., Lopez-Berestein G., Calin G.A. (2016). microRNA Therapeutics in Cancer—An Emerging Concept. EBioMedicine.

[144] Calin G.A., Cimmino A., Fabbri M., Ferracin M., Wojcik S.E., Shimizu M., Taccioli C., Zanesi N., Garzon R., Aqeilan R.I. (2008). MiR-15a and miR-16-1 cluster functions in human leukemia. Proc. Natl. Acad. Sci. USA.

[145] Cimmino A., Calin G.A., Fabbri M., Iorio M.V., Ferracin M., Shimizu M., Wojcik S.E., Aqeilan R.I., Zupo S., Dono M. (2005). miR-15 and miR-16 induce apoptosis by targeting BCL2. Proc. Natl. Acad. Sci. USA.

[146] Arun G., Diermeier S.D., Spector D.L. (2018). Therapeutic Targeting of Long Non-Coding RNAs in Cancer. Trends Mol. Med..

[147] Modarresi F., Faghihi M.A., A Lopez-Toledano M., Fatemi R.P., Magistri M., Brothers S.P., van der Brug M.P., Wahlestedt C. (2012). Inhibition of natural antisense transcripts in vivo results in gene-specific transcriptional upregulation. Nat. Biotechnol..

[148] Hsiao J., Yuan T., Tsai M., Lu C., Lin Y., Lee M., Lin S., Chang F., Pimentel H.L., Olive C. (2016). Upregulation of Haploinsufficient Gene Expression in the Brain by Targeting a Long Non-coding RNA Improves Seizure Phenotype in a Model of Dravet Syndrome. EBioMedicine.

[149] Padmakumar S., Jones G., Pawar G., Khorkova O., Hsiao J., Kim J., Amiji M.M., Bleier B.S. (2021). Minimally Invasive Nasal Depot (MIND) technique for direct BDNF AntagoNAT delivery to the brain. J. Control. Release.

[150] Moulder S.L., Symmans W.F., Booser D.J., Madden T.L., Lipsanen C., Yuan L., Brewster A.M., Cristofanilli M., Hunt K.K., Buchholz T.A. (2008). Phase I/II Study of G3139 (Bcl-2 Antisense Oligonucleotide) in Combination with Doxorubicin and Docetaxel in Breast Cancer. Clin. Cancer Res..

[151] Croce C.M., Reed J.C. (2016). Finally, An Apoptosis-Targeting Therapeutic for Cancer. Cancer Res..

[152] Gallant-Behm C.L., Piper J., Lynch J.M., Seto A.G., Hong S.J., Mustoe T.A., Maari C., Pestano L.A., Dalby C.M., Jackson A.L. (2018). A MicroRNA-29 Mimic (Remlarsen) Represses Extracellular Matrix Expression and Fibroplasia in the Skin. J. Investig. Dermatol..

[153] Chakraborty C., Sharma A.R., Sharma G., Bhattacharya M., Lee S.S. (2020). SARS-CoV-2 causing pneumonia-associated respiratory disorder (COVID-19): Diagnostic and proposed therapeutic options. Eur. Rev. Med. Pharmacol. Sci..

[154] Roberts T.C., Langer R., Wood M.J.A. (2021). Advances in oligonucleotide drug delivery. Nat. Rev. Drug Discov..

[155] Hu Y., Matkovich S.J., Hecker P.A., Zhang Y., Edwards J.R., Dorn G.W. (2012). Epitranscriptional orchestration of genetic reprogramming is an emergent property of stress-regulated cardiac microRNAs. Proc. Natl. Acad. Sci. USA.

[156] Bagnall R.D., Tsoutsman T., Shephard R.E., Ritchie W., Semsarian C. (2012). Global MicroRNA Profiling of the Mouse Ventricles during Development of Severe Hypertrophic Cardiomyopathy and Heart Failure. PLoS ONE.

[157] Raso A., Dirkx E., Sampaio-Pinto V., el Azzouzi H., Cubero R.J., Sorensen D.W., Ottaviani L., Olieslagers S., Huibers M.M., de Weger R. (2021). A microRNA program regulates the balance between cardiomyocyte hyperplasia and hypertrophy and stimulates cardiac regeneration. Nat. Commun..

[158] Gallo A., Agnese V., Sciacca S., Scardulla C., Cipriani M., Pilato M., Oh J.K., Pasta S., Maalouf J., Conaldi P.G. (2024). MicroRNA-30d and -483-3p for bi-ventricular remodelling and miR-126-3p for pulmonary hypertension in advanced heart failure. ESC Heart Fail..

[159] Ray K.K., Wright R.S. (2021). Clinical Update on Novel Lipid-Lowering Therapies to Reduce Cardiovascular Risk. JAMA.

[160] Janssen H.L.A., Reesink H.W., Lawitz E.J., Zeuzem S., Rodriguez-Torres M., Patel K., Van Der Meer A.J., Patick A.K., Chen A., Zhou Y. (2013). Treatment of HCV Infection by Targeting MicroRNA. N. Engl. J. Med..

[161] Sioud M. (2006). Single-stranded small interfering RNA are more immunostimulatory than their double-stranded counterparts: A central role for 2′-hydroxyl uridines in immune responses. Eur. J. Immunol..

[162] Morrissey D.V., A Lockridge J., Shaw L., Blanchard K., Jensen K., Breen W., Hartsough K., Machemer L., Radka S., Jadhav V. (2005). Potent and persistent in vivo anti-HBV activity of chemically modified siRNAs. Nat. Biotechnol..

[163] Judge A.D., Bola G., Lee A.C., MacLachlan I. (2006). Design of Noninflammatory Synthetic siRNA Mediating Potent Gene Silencing in Vivo. Mol. Ther..

[164] Liu C., Kelnar K., Liu B., Chen X., Calhoun-Davis T., Li H., Patrawala L., Yan H., Jeter C., Honorio S. (2011). The microRNA miR-34a inhibits prostate cancer stem cells and metastasis by directly repressing CD44. Nat. Med..

[165] Bader A.G. (2012). miR-34—A microRNA replacement therapy is headed to the clinic. Front. Genet..

[166] Daige C.L., Wiggins J.F., Priddy L., Nelligan-Davis T., Zhao J., Brown D. (2014). Systemic Delivery of a miR34a Mimic as a Potential Therapeutic for Liver Cancer. Mol. Cancer Ther..

[167] Tolcher A.W., Rodrigueza W.V., Rasco D.W., Patnaik A., Papadopoulos K.P., Amaya A., Moore T.D., Gaylor S.K., Bisgaier C.L., Sooch M.P. (2013). A phase 1 study of the BCL2-targeted deoxyribonucleic acid inhibitor (DNAi) PNT2258 in patients with advanced solid tumors. Cancer Chemother. Pharmacol..

[168] Kelnar K., Bader A.G. (2015). A qRT-PCR Method for Determining the Biodistribution Profile of a miR-34a Mimic. Methods Mol. Biol..

[169] Wang X., Li J., Dong K., Lin F., Long M., Ouyang Y., Wei J., Chen X., Weng Y., He T. (2015). Tumor suppressor miR-34a targets PD-L1 and functions as a potential immunotherapeutic target in acute myeloid leukemia. Cell. Signal..

[170] Täubel J., Hauke W., Rump S., Viereck J., Batkai S., Poetzsch J., Rode L., Weigt H., Genschel C., Lorch U. (2020). Novel antisense therapy targeting microRNA-132 in patients with heart failure: Results of a first-in-human Phase 1b randomized, double-blind, placebo-controlled study. Eur. Heart J..

[171] Hinkel R., Batkai S., Bähr A., Bozoglu T., Straub S., Borchert T., Viereck J., Howe A., Hornaschewitz N., Oberberger L. (2021). AntimiR-132 attenuates myocardial hypertrophy in an animal model of percutaneous aortic constriction. J. Am. Coll. Cardiol..

[172] Van Der Ree M.H., Van Der Meer A.J., De Bruijne J., Maan R., Van Vliet A., Welzel T.M., Zeuzem S., Lawitz E.J., Rodriguez-Torres M., Kupcova V. (2014). Long-term safety and efficacy of microRNA-targeted therapy in chronic hepatitis C patients. Antivir. Res..

[173] Van der Ree M.H., Van Der Meer A.J., Van Nuenen A.C., De Bruijne J., Ottosen S., Janssen H.L., Kootstra N.A., Reesink H.W. (2016). Miravirsen dosing in chronic hepatitis C patients results in decreased microRNA-122 levels without affecting other microRNAs in plasma. Aliment. Pharmacol. Ther..

[174] Natarelli L., Geißler C., Csaba G., Wei Y., Zhu M., di Francesco A., Hartmann P., Zimmer R., Schober A. (2018). miR-103 promotes endothelial maladaptation by targeting lncWDR59. Nat. Commun..

[175] Deng Y., Campbell F., Han K., Theodore D., Deeg M., Huang M., Hamatake R., Lahiri S., Chen S., Horvath G. (2020). Randomized clinical trials towards a single-visit cure for chronic hepatitis C: Oral GSK2878175 and injectable RG-101 in chronic hepatitis C patients and long-acting injectable GSK2878175 in healthy participants. J. Viral Hepat..

[176] van der Ree M.H., de Vree J.M., Stelma F., Willemse S., van der Valk M., Rietdijk S., Molenkamp R., Schinkel J., Van Nuenen A.C., Beuers U. (2017). Safety, tolerability, and antiviral effect of RG-101 in patients with chronic hepatitis C: A phase 1B, double-blind, randomised controlled trial. Lancet.

[177] Nappi F., Spadaccio C., Al-Attar N., Acar C. (2015). The Ross procedure at the crossroads: Lessons from biology: Is Dr Ross’s dream concluded?. Int. J. Cardiol..

[178] Lee E.C., Valencia T., Allerson C., Schairer A., Flaten A., Yheskel M., Kersjes K., Li J., Gatto S., Takhar M. (2019). Discovery and preclinical evaluation of anti-miR-17 oligonucleotide RGLS4326 for the treatment of polycystic kidney disease. Nat. Commun..

[179] Du W., Pan Z., Chen X., Wang L., Zhang Y., Li S., Liang H., Xu C., Zhang Y., Wu Y. (2014). By Targeting Stat3 microRNA-17-5p Promotes Cardiomyocyte Apoptosis in Response to Ischemia Followed by Reperfusion. Cell. Physiol. Biochem..

[180] Anastasiadou E., Seto A.G., Beatty X., Hermreck M., Gilles M.-E., Stroopinsky D., Pinter-Brown L.C., Pestano L., Marchese C., Avigan D. (2021). Cobomarsen, an Oligonucleotide Inhibitor of miR-155, Slows DLBCL Tumor Cell Growth In Vitro and In Vivo. Clin. Cancer Res..

[181] Abplanalp W.T., Fischer A., John D., Zeiher A.M., Gosgnach W., Darville H., Montgomery R., Pestano L., Allée G., Paty I. (2020). Efficiency and target derepression of anti-miR-92a: Results of a first in human study. Nucleic Acid. Ther..

[182] Yang Y., Cheng H.W., Qiu Y., Dupee D., Noonan M., Lin Y.D., Fisch S., Unno K., Sereti K.I., Liao R. (2015). MicroRNA-34a Plays a Key Role in Cardiac Repair and Regeneration Following Myocardial Infarction. Circ. Res..

[183] Gabisonia K., Prosdocimo G., Aquaro G.D., Carlucci L., Zentilin L., Secco I., Ali H., Braga L., Gorgodze N., Bernini F. (2019). MicroRNA therapy stimulates uncontrolled cardiac repair after myocardial infarction in pigs. Nature.

[184] Juliano R.L. (2016). The delivery of therapeutic oligonucleotides. Nucleic Acids Res..

[185] Geary R.S., Norris D., Yu R., Bennett C.F. (2015). Pharmacokinetics, biodistribution and cell uptake of antisense oligonucleotides. Adv. Drug Deliv. Rev..

[186] Eding J.E., Demkes C.J., Lynch J.M., Seto A.G., Montgomery R.L., Semus H.M., Jackson A.L., Isabelle M., Chimenti S., van Rooij E. (2017). The Efficacy of Cardiac Anti-miR-208a Therapy Is Stress Dependent. Mol. Ther..

[187] Stein C.A., Hansen J.B., Lai J., Wu S., Voskresenskiy A., Høg A., Worm J., Hedtjärn M., Souleimanian N., Miller P. (2010). Efficient gene silencing by delivery of locked nucleic acid antisense oligonucleotides, unassisted by transfection reagents. Nucleic Acids Res..

[188] Hullinger T.G., Montgomery R.L., Seto A.G., Dickinson B.A., Semus H.M., Lynch J.M., Dalby C.M., Robinson K., Stack C., Latimer P.A. (2012). Inhibition of miR-15 protects against cardiac ischemic injury. Circ. Res..

[189] Icli B., Wara A., Moslehi J., Sun X., Plovie E., Cahill M., Marchini J.F., Schissler A., Padera R.F., Shi J. (2013). MicroRNA-26a Regulates Pathological and Physiological Angiogenesis by Targeting BMP/SMAD1 Signaling. Circ. Res..

[190] Tijsen A.J., van der Made I., Hoogenhof M.M.v.D., Wijnen W.J., van Deel E.D., de Groot N.E., Alekseev S., Fluiter K., Schroen B., Goumans M.-J. (2014). The microRNA-15 family inhibits the TGFβ-pathway in the heart. Cardiovasc. Res..

[191] Yang F., Chen Q., He S., Yang M., Maguire E.M., An W., Afzal T.A., Luong L.A., Zhang L., Xiao Q. (2018). miR-22 Is a Novel Mediator of Vascular Smooth Muscle Cell Phenotypic Modulation and Neointima Formation. Circulation.

[192] Dirkx E., Gladka M.M., Philippen L.E., Armand A.-S., Kinet V., Leptidis S., el Azzouzi H., Salic K., Bourajjaj M., da Silva G.J.J. (2013). Nfat and miR-25 cooperate to reactivate the transcription factor Hand2 in heart failure. Nature.

[193] Rayner K.J., Sheedy F.J., Esau C.C., Hussain F.N., Temel R.E., Parathath S., van Gils J.M., Rayner A.J., Chang A.N., Suarez Y. (2011). Antagonism of miR-33 in mice promotes reverse cholesterol transport and regression of atherosclerosis. J. Clin. Investig..

[194] Mitchell M.J., Billingsley M.M., Haley R.M., Wechsler M.E., Peppas N.A., Langer R. (2020). Engineering precision nanoparticles for drug delivery. Nat. Rev. Drug Discov..

[195] Domenger C., Grimm D. (2019). Next-generation AAV vectors—Do not judge a virus (only) by its cover. Hum. Mol. Genet..

[196] Li C., Samulski R.J. (2020). Engineering adeno-associated virus vectors for gene therapy. Nat. Rev. Genet..

[197] Zeng Y., Du W.W., Wu Y., Yang Z., Awan F.M., Li X., Yang W., Zhang C., Yang Q., Chen Y. (2017). A Circular RNA Binds To and Activates AKT Phosphorylation and Nuclear Localization Reducing Apoptosis and Enhancing Cardiac Repair. Theranostics.

[198] Gruner H.N., McManus M.T. (2021). Examining the evidence for extracellular RNA function in mammals. Nat. Rev. Genet..

[199] Sahoo S., Adamiak M., Mathiyalagan P., Kenneweg F., Kafert-Kasting S., Thum T. (2021). Therapeutic and diagnostic translation of extracellular vesicles in cardiovascular diseases: Roadmap to the clinic. Circulation.

[200] Chakraborty R., Saddouk F.Z., Carrao A.C., Krause D.S., Greif D.M., Martin K.A. (2019). Promoters to Study Vascular Smooth Muscle. Arterioscler. Thromb. Vasc. Biol..

[201] Bian J., Popovic Z.B., Benejam C., Kiedrowski M., Rodriguez L.L., Penn M.S. (2007). Effect of cell-based intercellular delivery of transcription factor GATA4 on ischemic cardiomyopathy. Circ. Res..

[202] Zahid M., Feldman K.S., Garcia-Borrero G., Feinstein T.N., Pogodzinski N., Xu X., Yurko R., Czachowski M., Wu Y.L., Mason N.S. (2018). Cardiac Targeting Peptide, a Novel Cardiac Vector: Studies in Bio-Distribution, Imaging Application, and Mechanism of Transduction. Biomolecules.

[203] Grijalvo S., Alagia A., Jorge A.F., Eritja R. (2018). Covalent Strategies for Targeting Messenger and Non-Coding RNAs: An Updated Review on siRNA, miRNA and antimiR Conjugates. Genes.

[204] Sugo T., Terada M., Oikawa T., Miyata K., Nishimura S., Kenjo E., Ogasawara-Shimizu M., Makita Y., Imaichi S., Murata S. (2016). Development of antibody-siRNA conjugate targeted to cardiac and skeletal muscles. J. Control. Release.

[205] Klein D., Goldberg S., Theile C.S., Dambra R., Haskell K., Kuhar E., Lin T., Parmar R., Manoharan M., Richter M. (2021). Centyrin ligands for extrahepatic delivery of siRNA. Mol. Ther..

[206] Orellana E.A., Tenneti S., Rangasamy L., Lyle L.T., Low P.S., Kasinski A.L. (2017). FolamiRs: Ligand-targeted, vehicle-free delivery of mi-croRNAs for the treatment of cancer. Sci. Transl. Med..

[207] Bom A.P.D.A., Neves P.C.d.C., de Almeida C.E.B., Silva D., Missailidis S. (2019). Aptamers as Delivery Agents of siRNA and Chimeric Formulations for the Treatment of Cancer. Pharmaceutics.

[208] Rohde J.-H., Weigand J.E., Suess B., Dimmeler S. (2015). A Universal Aptamer Chimera for the Delivery of Functional microRNA-126. Nucleic Acid Ther..

[209] Asokan A., Conway J.C., Phillips J.L., Li C., Hegge J., Sinnott R., Yadav S., DiPrimio N., Nam H.-J., Agbandje-McKenna M. (2010). Reengineering a receptor footprint of adeno-associated virus enables selective and systemic gene transfer to muscle. Nat. Biotechnol..

[210] Ishikawa K., Fish K.M., Tilemann L., Rapti K., Aguero J., Santos-Gallego C.G., Lee A., Karakikes I., Xie C., Akar F.G. (2014). Cardiac I-1c Overexpression With Reengineered AAV Improves Cardiac Function in Swine Ischemic Heart Failure. Mol. Ther..

[211] Tabebordbar M., Lagerborg K.A., Stanton A., King E.M., Ye S., Tellez L., Krunnfusz A., Tavakoli S., Widrick J.J., Messemer K.A. (2021). Directed evolution of a family of AAV capsid variants enabling potent muscle-directed gene delivery across species. Cell.

[212] Duygu B., Juni R., Ottaviani L., Bitsch N., Wit J.B., de Windt L.J., Martins P.A.d.C. (2018). Comparison of different chemically modified inhibitors of miR-199b in vivo. Biochem. Pharmacol..

[213] Raso A., Dirkx E., Philippen L.E., Fernandez-Celis A., De Majo F., Sampaio-Pinto V., Sansonetti M., Juni R., el Azzouzi H., Calore M. (2018). Therapeutic Delivery of miR-148a Suppresses Ventricular Dilation in Heart Failure. Mol. Ther..

[214] Yang H., Qin X., Wang H., Zhao X., Liu Y., Wo H.-T., Liu C., Nishiga M., Chen H., Ge J. (2019). An *in Vivo* miRNA Delivery System for Restoring Infarcted Myocardium. ACS Nano.

[215] Rayner K.J., Esau C.C., Hussain F.N., McDaniel A.L., Marshall S.M., van Gils J.M., Ray T.D., Sheedy F.J., Goedeke L., Liu X. (2011). Inhibition of miR-33a/b in non-human primates raises plasma HDL and lowers VLDL triglycerides. Nature.

[216] van Meer L., Moerland M., Gallagher J., van Doorn M.B.A., Prens E.P., Cohen A.F., Rissmann R., Burggraaf J. (2016). Injection site reactions after subcutaneous oligonucleotide therapy. Br. J. Clin. Pharmacol..

[217] Rogg E.-M., Abplanalp W.T., Bischof C., John D., Schulz M.H., Krishnan J., Fischer A., Poluzzi C., Schaefer L., Bonauer A. (2018). Analysis of Cell Type-Specific Effects of MicroRNA-92a Provides Novel Insights Into Target Regulation and Mechanism of Action. Circulation.

[218] Wahlquist C., Jeong D., Rojas-Muñoz A., Kho C., Lee A., Mitsuyama S., van Mil A., Park W.J., Sluijter J.P.G., Doevendans P.A.F. (2014). Inhibition of miR-25 improves cardiac contractility in the failing heart. Nature.

[219] A Dobrovolskaia M., E McNeil S. (2015). Immunological and hematological toxicities challenging clinical translation of nucleic acid-based therapeutics. Expert Opin. Biol. Ther..

[220] Judge A.D., Sood V., Shaw J.R., Fang D., McClintock K., MacLachlan I. (2005). Sequence-dependent stimulation of the mammalian innate immune response by synthetic siRNA. Nat. Biotechnol..

[221] Karikó K., Buckstein M., Ni H., Weissman D. (2005). Suppression of RNA Recognition by Toll-like Receptors: The Impact of Nucleoside Modification and the Evolutionary Origin of RNA. Immunity.

[222] Broering R., Real C.I., John M.J., Jahn-Hofmann K., Ickenstein L.M., Kleinehr K., Paul A., Gibbert K., Dittmer U., Gerken G. (2014). Chemical modifications on siRNAs avoid Toll-like-receptor-mediated activation of the hepatic immune system in vivo and in vitro. Int. Immunol..

[223] Alharbi A.S., Garcin A.J., A Lennox K., Pradeloux S., Wong C., Straub S., Valentin R., Pépin G., Li H.-M., Nold M.F. (2020). Rational design of antisense oligonucleotides modulating the activity of TLR7/8 agonists. Nucleic Acids Res..

[224] Povsic T.J., Lawrence M.G., Lincoff A.M., Mehran R., Rusconi C.P., Zelenkofske S.L., Huang Z., Sailstad J., Armstrong P.W., Steg P.G. (2016). Pre-existing anti-PEG antibodies are associated with severe immediate allergic reactions to pegnivacogin, a PEGylated aptamer. J. Allergy Clin. Immunol..

[225] Shen W., De Hoyos C.L., Migawa M.T., Vickers T.A., Sun H., Low A., Bell T.A., Rahdar M., Mukhopadhyay S., Hart C.E. (2019). Chemical modification of PS-ASO therapeutics reduces cellular protein-binding and improves the therapeutic index. Nat. Biotechnol..

[226] Androsavich J.R., Chau B.N. (2014). Non-inhibited miRNAs shape the cellular response to anti-miR. Nucleic Acids Res..

[227] A Khan A., Betel D., Miller M.L., Sander C., Leslie C.S., Marks D.S. (2009). Transfection of small RNAs globally perturbs gene regulation by endogenous microRNAs. Nat. Biotechnol..

[228] Qu C., Cui H., Xiao S., Dong L., Lu Q., Zhang L., Wang P., Xin M., Zhi H., Liu C. (2024). The landscape of immune checkpoint-related long non-coding RNAs core regulatory circuitry reveals implications for immunoregulation and immunotherapy responses. Commun. Biol..

[229] de Boer R.A., Hulot J., Tocchetti C.G., Aboumsallem J.P., Ameri P., Anker S.D., Bauersachs J., Bertero E., Coats A.J., Čelutkienė J. (2020). Common mechanistic pathways in cancer and heart failure. A scientific roadmap on behalf of the Translational Research Committee of the Heart Failure Association (HFA) of the European Society of Cardiology (ESC). Eur. J. Heart Fail..

[230] Sahraei M., Chaube B., Liu Y., Sun J., Kaplan A., Price N.L., Ding W., Oyaghire S., García-Milian R., Mehta S. (2019). Suppressing miR-21 activity in tumor-associated macrophages promotes an antitumor immune response. J. Clin. Investig..

[231] Mastroianni J., Stickel N., Andrlova H., Hanke K., Melchinger W., Duquesne S., Schmidt D., Falk M., Andrieux G., Pfeifer D. (2019). miR-146a Controls Immune Response in the Melanoma Microenvironment. Cancer Res.

[232] Mu P., Han Y.-C., Betel D., Yao E., Squatrito M., Ogrodowski P., de Stanchina E., D’Andrea A., Sander C., Ventura A. (2009). Genetic dissection of the *miR-17∼92* cluster of microRNAs in Myc-induced B-cell lymphomas. Genes Dev..

[233] Kilikevicius A., Meister G., Corey D.R. (2021). Reexamining assumptions about miRNA-guided gene silencing. Nucleic Acids Res..

[234] Dong C., Hui P., Wu Z., Li J., Man X. (2024). CircRNA LOC729852 promotes bladder cancer progression by regulating macrophage polarization and recruitment via the miR-769-5p/IL-10 axis. J. Cell. Mol. Med..

[235] Rybak-Wolf A., Stottmeister C., Glažar P., Jens M., Pino N., Giusti S., Hanan M., Behm M., Bartok O., Ashwal-Fluss R. (2015). Circular RNAs in the Mammalian Brain Are Highly Abundant, Conserved, and Dynamically Expressed. Mol. Cell.

[236] Kristensen L.S., Andersen M.S., Stagsted L.V.W., Ebbesen K.K., Hansen T.B., Kjems J. (2019). The biogenesis, biology and characterization of circular RNAs. Nat. Rev. Genet..

[237] Bachmayr-Heyda A., Reiner A.T., Auer K., Sukhbaatar N., Aust S., Bachleitner-Hofmann T., Mesteri I., Grunt T.W., Zeillinger R., Pils D. (2015). Correlation of circular RNA abundance with proliferation–exemplified with colorectal and ovarian cancer, idiopathic lungfibrosis, and normal human tissues. Sci. Rep..

[238] Chen Y.G., Kim M.V., Chen X., Batista P.J., Aoyama S., Wilusz J.E., Iwasaki A., Chang H.Y. (2017). Sensing Self and Foreign Circular RNAs by Intron Identity. Mol. Cell.

[239] Chen Y.G., Chen R., Ahmad S., Verma R., Kasturi S.P., Amaya L., Broughton J.P., Kim J., Cadena C., Pulendran B. (2019). N6-Methyladenosine Modification Controls Circular RNA Immunity. Mol. Cell.

[240] Wesselhoeft R.A., Kowalski P.S., Parker-Hale F.C., Huang Y., Bisaria N., Anderson D.G. (2019). RNA circularization diminishes immunogenicity and can extend translation duration in vivo. Mol. Cell.

